# Design, Synthesis, and Biological Activity of Novel Myricetin Derivatives Containing Amide, Thioether, and 1,3,4-Thiadiazole Moieties

**DOI:** 10.3390/molecules23123132

**Published:** 2018-11-29

**Authors:** Xianghui Ruan, Cheng Zhang, Shichun Jiang, Tao Guo, Rongjiao Xia, Ying Chen, Xu Tang, Wei Xue

**Affiliations:** Key Laboratory of Green Pesticide and Agriculture Bioengineering, Ministry of Education, Guizhou University, Huaxi District, Guiyang 550025, China; rxh9105@163.com (X.R.); zhangcheng6954@163.com (C.Z.); 15885526512@163.com (S.J.); guotao9405163@163.com (T.G.); xrjiao@163.com (R.X.); 15286114381@163.com (Y.C.); tx1173182020@163.com (X.T.)

**Keywords:** myricetin, amide, 1,3,4-oxadiazole, thioether, bioactivity

## Abstract

A series of myricetin derivatives containing amide, thioether, and 1,3,4-thiadiazole moieties were designed and synthesized, and their antiviral and antibacterial activities were assessed. The bioassays showed that all the title compounds exhibited potent in vitro antibacterial activities against *Xanthomonas citri* (*Xac*), *Ralstonia solanacearum* (*Rs*), and *Xanthomonas oryzae pv. Oryzae* (*Xoo*). In particular, the compounds **5a**, **5f**, **5g**, **5h**, **5i,** and **5l**, with EC_50_ values of 11.5–27.3 μg/mL, showed potent antibacterial activity against *Xac* that was better than the commercial bactericides Bismerthiazol (34.7 μg/mL) and Thiodiazole copper (41.1% μg/mL). Moreover, the in vivo antiviral activities against tobacco mosaic virus (TMV) of the target compounds were also tested. Among these compounds, the curative, protection, and inactivation activities of **5g** were 49.9, 52.9, and 73.3%, respectively, which were better than that of the commercial antiviral Ribavirin (40.6, 51.1, and 71.1%, respectively). This study demonstrates that myricetin derivatives bearing amide, thioether, and 1,3,4-thiadiazole moieties can serve as potential alternative templates for the development of novel, highly efficient inhibitors against plant pathogenic bacteria and viruses.

## 1. Introduction

In recent years, pathogenic bacteria and plant viruses targeting agricultural crops have become increasingly difficult to control, leading to enormous losses in worldwide crop production each year. As such, the development of environmentally friendly pesticides with high selectivity, high efficiency, low toxicity, and suitable degradation are important challenges to both chemists and biologists [1]. Natural products and extracts, derived from the biosphere, have been widely applied for use in medicines [2], pesticides [3,4], cosmetics [5], and food products in ancient and modern societies around the world [6]. Recently, many natural products have shown promise as antimicrobials and antivirals [7], including the quercetin, genistein, and silybin, which belong to the flavonoids group of compounds and have demonstrated inhibitory activity against the DNA virus herpes type 1 (HSV-1) and RNA virus parainfluenza type 3 (PI-3) [8]. Consequently, our research has focused on the properties of natural products as a starting point for the design of pesticide molecules.

**Figure 1:**
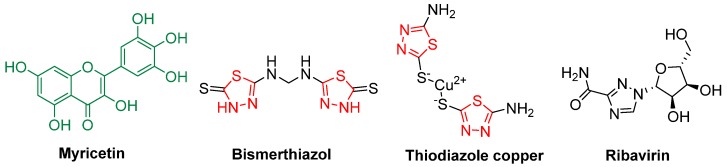
The structures of myricetin and selected commercial drugs.

Myricetin (Figure 1) belongs to a class of natural flavonoids that is widely found in vegetables, fruits, teas, and medicine herbs [9,10]. Over the last decade, many researchers have shown that natural myricetin exhibits various biological activities, including antibacterial [11], antiviral [12], antioxidant [9,13], anti-inflammatory [14], and anticancer [15,16,17] properties. Most of these prior studies have assessed the properties of myricetin itself, but its derivatives have rarely been studied [18,19,20,21]. Recently, in our prior work, some myricetin derivatives bearing 1,3,4-thiadiazole [18], Schiff-base [20], and acid amide [21] moieties were rationally synthesized and assessed for their potential application as novel anticarcinogens and bactericides.

The compound 1,3,4-thiadiazole contains an important nitrogenous pharmacophore and exhibits different biological activities, such as antimicrobial, antidepressant, and anti-tumor activities. The 1,3,4-thiadiazole ring is very weak due to the induction of sulfur atoms, which makes it easy for it to unlink in alkaline aqueous solutions; however, it is relatively stable in acidic aqueous solutions [22]. The ring readily reacts with a nucleophile to generate diverse derivatives due to its electronic state [23]. Commercial bactericides and antivirals bearing 1,3,4-thiadiazoles have been developed and are widely available, and these include the agricultural bactericidal agents Bismerthiazol and Thiodiazole copper (Figure 1).

Based on these considerations, we designed and synthesized a series of novel myricetin derivatives containing amide, thioether, and 1,3,4-thiadiazole moieties. The derivatives were evaluated via turbidimetry tests and screened against three plant pathogens *Xanthomonas citri* (*Xac*), *Ralstonia solanacearum* (*Rs*), and *Xanthomonas oryzae pv. Oryzae* (*Xoo*). In addition, the ability of these compounds to inhibit tobacco mosaic virus (TMV) was also evaluated in vivo via the half-leaf method at 500 μg/mL, with the commercial antiviral Ribavirin serving as a positive control (Figure 1).

## 2. Results and Discussion

### 2.1. Chemistry

**Scheme 1:**
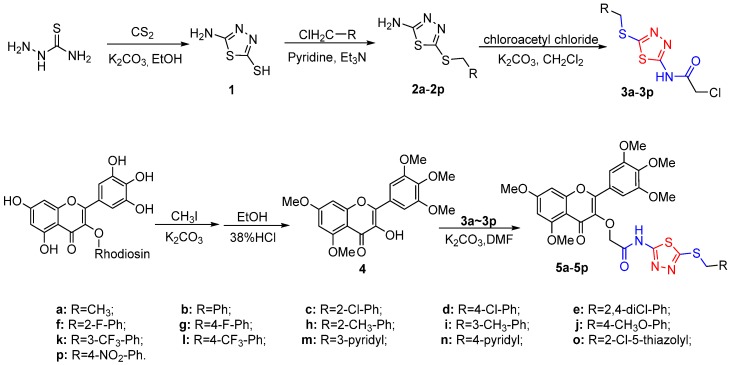
Synthesis of the title compounds **5a**–**5p**.

The synthesis protocol was divided into five steps (Scheme 1). Firstly, 2-amino-5-mercapto-1,3,4-thiadiazole (intermediate **1**) was obtained according to the previously reported method [24]. Secondly, intermediates **2a**–**2p** were synthesized from intermediate **1** and various benzyl chlorides via substitution reaction. Thirdly, the intermediates **3a**–**3p** were synthesized via a substitution reaction by treating intermediates **2a**–**2p** with chloroacetyl chloride in the presence of the base K_2_CO_3_. Fourthly, myricitrin was converted to intermediate **4** by methylation and deglycosidation. Finally, the 3-OH on intermediate **4** underwent a substitution reaction with the halogen atom of intermediates **3a**–**3p** to generate the target compounds **5a**–**5p**.

The structures of all the target compounds (**5a**–**5p**) were verified by ^1^H-NMR, ^13^C-NMR, and HRMS. The spectra data are provided in the experiments below. The ^1^H-NMR results showed that all the aryl protons presented as multiplets at approximately 8.65–6.40 ppm, and the characteristic -O-CH_2_- and -S-CH_2_- groups between the myricetin scaffold and 1,3,4-thiadiazole heterocycle were observed at approximately 4.36–4.91 ppm. In the ^13^C-NMR spectra, the typical shifts near 165–169, 150–155, and 31–32 ppm reveal the presence of -N=C- and C=O, the carbon atoms of the 1,3,4-thiadiazole, and -S-CH_2_- groups.

### 2.2. Antiviral Activities of the Title Compounds against Plant Pathogens In Vivo

Antibacterial activities of myricetin derivatives containing the amide, thioether, and 1,3,4-oxadiazole moieties (**5a**–**5p**) against the plant pathogens *Xac*, *Rs,* and *Xoo* were evaluated in vitro using a turbidimeter method [25,26,27]. The standard drug Myricetin (MYR) and the commercial bactericides Thiodiazole copper (TC) and Bismerthiazol (BT) were chosen as control agents. The results are shown in Table 1. Most of the target compounds exhibited similar or slightly enhanced inhibition activities against the pathogens *Xac*, *Rs,* and *Xoo* at the two concentrations tested (100 and 50 μg/mL).

Among the title compounds, the inhibitory rates of compounds **5a**, **5f**, **5g**, **5h**, **5i,** and **5l** possessed excellent antibacterial activities against *Xac* at 100 μg/mL were 75.7, 75.0, 89.2, 74.3, 89.5, and 75.3%, respectively, which were better than those of the commercial bactericides BT (73.3%) and TC (67.7%). The activities of the above target compounds against *Xac* at 50 μg/mL were 64.3, 60.3, 79.5, 68.3, 68.7, and 55.3% respectively, which were better than those of BT (55.0%) and TC (44.3%). Moreover, the inhibition rates of compounds **5a**, **5e,** and **5i** at 100 μg/mL against *Rs* were 70.4, 68.7, 78.0, and 85.6%, respectively, which exceeded that of the BT (69.7%). Compounds **5c**, **5e,** and **5i** at 50 μg/mL showed 63.4, 60.5, and 64.8% inhibition rates, respectively, which were superior to that of BT (57.6%). Furthermore, the inhibitory activities of all the target compounds at 100 μg/mL against *Xoo* were better than the BT (71.1%) and similar to TC (36.1%). Notably, compound **5i** showed comparable or stronger antibacterial activities against *Xac*, *Rs,* and *Xoo* when compared with the two commercial bactericides. Thus, compound **5i** was regarded as the most promising antibacterial agent and used for further evaluation. According to our previous work on myricetin derivatives containing 1,3,4-thiadiazole [18], when the R group is CH_3_, Ph, 2-CH_3_-Ph, and 4-CH_3_O-Ph, the antibacterial activities against *Xoo* of our present work is better than previous work, when R group is 2,4-diCl-Ph, 4-CH_3_O-Ph, and 2-Cl-thiazolyl, the antibacterial activities against *Rs* is better than previously.

Based on previously described bioassays methods [28], some of the compounds with excellent antibacterial activities were tested at five halved concentrations (100, 50, 25, 12.5, and 6.25 μg/mL) in order to obtain their corresponding EC_50_ values. The screening results are shown in Table 2. All of the tested compounds exhibited better antibacterial activities against the pathogens *Xac* and *Rs* when compared with BT and TC. In addition, compounds **5e**, **5f,** and **5i** exhibited EC_50_ values of 34.0, 44.9, and 31.2 μg/mL, respectively, which were better than BT and TC (45.3 and 105.4 μg/mL, respectively). Compared with previous work [18], when the R group is CH_3_, 2-Cl-Ph, 2,4-diCl-Ph, and 1,3,4-thiadiazole myricetin derivatives exhibited excellent inhibitory effect on *Rs* at low concentrations.

### 2.3. Antiviral Activities of the Title Compounds against TMV In Vivo

At the concentration of 500 μg/mL, the antiviral activities of the target compounds **5a**−**5p** were tested in vivo against TMV using the half-leaf method [7,29], and the results are listed in Table 3. For comparison, the commercial antiviral agent ribavirin (RBV) was chosen as the positive control for TMV. The results indicated that compounds **5a**–**5p** showed various degrees of antiviral activities against TMV. Some of these compounds displayed promising curative and inactivation activities, with curative and inactivation rates ranging from 22.3 to 49.9% and 51.5 to 76.7%, respectively. The compounds **5f**, **5g**, **5i**, **5l,** and **5n** showed 42.2, 49.9, 44.2, 45.3**,** and 43.1% curative effects at 500 μg/mL, respectively, which were better than that of RBV (40.6%). Compounds **5g** and **5j** with values of 52.9 and 54.5% showed protective activity against TMV. Moreover, the inactivation activities of all the compounds were similar to that of RBV (71.1%). Compared to our previous work on myricetin derivatives containing 1,3,4-thiadiazole [18], when the R group is Me or 2-F-Ph, the anti-TMV curative activities of present work is better than that of past work. When the R group is CH_3_, 2-F-Ph and 4-CH_3_O-Ph, the anti-TMV protective effect of this work is superior to that of the previous work.

**Figure 2:**
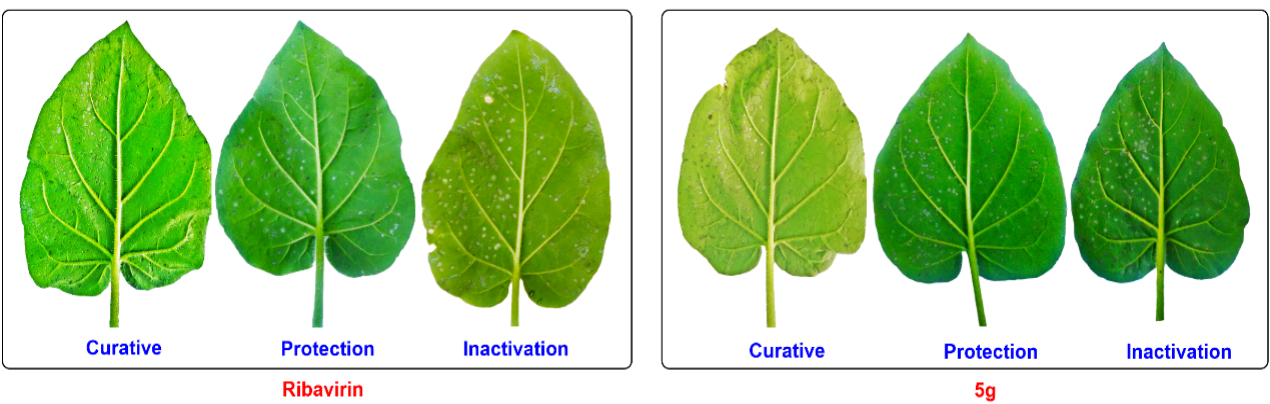
Tobacco leaf morphology effects of the RBV and **5g** against TMV in vivo. (Left leaf: Not treated with compound, right leaf: Smeared with compound).

Most target compounds possess better in curative, protective, and inactivation activities against TMV in vivo when compared to RBV. Remarkably, compound **5g** was superior to RBV in regard to all three activities and, thus, was noted as a promising lead compound. As Figure 2 shows, the number of local lesion on the leaf treated with either agent (RBV or **5g**) was significantly less than that without treatment. In addition, the area of the smeared with compound was smaller. Regarding the inhibitory mechanism of myricetin on TMV, we are studying it and will report it in subsequent studies. The inhibition rate of the compound was calculated according to the following formula. The average of three replicates were conducted for each sample.

Inhibition rate (%) = [(local lesion number of control (not treated with compound)—local lesion number smeared with compound)/ local lesion number of control ×100%.

## 3. Experimental

**Instruments.** The melting points (m.p.) of the title compounds were determined when left untouched on an XT-4-MP apparatus (Beijing Tech Instrument, Beijing, China). The ^1^H-NMR and ^13^C-NMR spectra were recorded on a JEOL-ECX 500 NMR spectrometer (JEOL, Tokyo, Japan) at ambient temperature operating at 500 MHz for ^1^H-NMR and 126 MHz for ^13^C-NMR, using CDCl_3_ or DMSO-*d*_6_ or (CD_3_)_2_CO as solvents and TMS as an internal standard. Chemical shifts were given in δ relative to TMS. The HRMS spectrogram was performed on a Thermo Scientific Q Exactive (Thermo, Waltham, MA, USA). Unless noted, all reagents were of analytical grade or chemically pure and purchased from Tansoole (Shanghai, China). All anhydrous solvents were dried and purified immediately prior to use in accordance with standard techniques.

### 3.1. Chemistry

#### 3.1.1. Synthesis Procedure for Intermediate **1**

Intermediate **1** was synthesized as detailed in a prior report [30]. A mixture of CS_2_ (120 mmol), anhydrous K_2_CO_3_ (50 mmol), and thiosemicarbazide (100 mmol) in absolute ethanol (120 mL) was stirred under reflux for one hour and then further heated on a steam bath for four hours. Upon completion of the reaction (as indicated by TLC), the solvent was removed, and the residue was dissolved in water, after dropwise addition of 10% HCl, to afford a white solid. The solid was filtered and washed with cold water. Then, the crude product was filtered, washed, and dried to obtain 5-amino-1,3,4-thiadiazole-2-thiol (1) 10.64 g with a 80% yield. m.p. 234–236 °C (References 233 °C [30]).

#### 3.1.2. General Procedure for Preparation of Intermediates **2a**–**6p**

Intermediate **2** was synthesized as detailed in a prior report [31] with minor modification. Intermediate **1** (3.8 mmol) and triethylamine (7.6 mmol) were added into 30 mL of pyridine. The mixture was incubated ambient temperature for 0.5 h. Then, bromoethane (3.9 mmol) was added to the reaction system and the mixture was treated at room temperature for four h. After the reaction was completed (as indicated by TLC), 100 mL of deionized water was added to the mixture and left to stand for 10 min. The mixture was then filtered, and the solid was washed with water and subsequently dried under vacuum. Intermediate **2a** was obtained 0.55 g at a yield of 89%. m.p. 136–137 °C. Intermediates **2b**–**2p** were prepared following a similar procedure.

#### 3.1.3. General Procedure for Preparation of Intermediates **3a**−**3p**

Compounds **3a**–**3p** were synthesized according to known methods [32]. K_2_CO_3_ (4.5 mmol) were added to a solution of intermediate **2a** (1.8 mmol) in dichloromethane (30 mL). The resulting mixture was then stirred at room temperature for 30 min. Next, a solution prepared by dissolving chloroacetyl chloride (2.7 mmol) in 5 mL dichloromethane was added to the reaction system in a drop wise fashion. The mixture was then stirred at ambient temperature for a further eight hours. After completion of the reaction, the mixture was refluxed for 30 min, and the solvent was removed under reduced pressure. Then, water (20 mL) was added to the mixture to afford a white solid, which was filtered and recrystallized from methanol/water (1:3/*v*:*v*). Intermediate **3a** was obtained 0.34 g with a yield of 77%. m.p. 166–168 °C. Following a similar procedure, intermediates **3b**–**3p** were prepared.

#### 3.1.4. General Procedure for Preparation of Intermediate **4**

Preparation of the intermediate **4** has been previously described [19]. The mixture of myricitrin (10 mmol), CH_3_I (130 mmol), and K_2_CO_3_ (12 mmol) was dissolved in DMF (60 mL), and stirred at 40 °C for two days until the reaction was complete (as indicated by TLC analysis). The reaction mixtures were then filtered, and the filtrate was dissolved in 100 mL water and finally extracted three times with dichloromethane (30 mL × 3), combined the dichloromethane, and concentrated under reduced pressure. The concentrated solution was diluted with 40 mL of absolute ethanol, stirred, and refluxed for one hour. The concentrated hydrochloric acid (9 mL) was slowly added to the above obtained for two hours in reflux. The solid was precipitated from the clear solution. After cooling to room temperature, the reaction mixture was filtered, and the obtained solid product was dried at 40 °C for two hours. Intermediate **4** was obtained 2.38 g at a yield of 75%. m.p. 228–230 °C (References 227–229 °C [19]).

#### 3.1.5. General Procedure for Preparation of Title Compounds **5a**−**5p**

K_2_CO_3_ (2.1 mmol) was added to a solution of intermediate **4** (0.7 mmol) in DMF (10 mL). The mixture was stirred at room temperature for 0.5 h. Then, a solution prepared by dissolving intermediate **3a** (0.7 mmol) in five mL DMF was added to the reaction system in a dropwise fashion. The reaction mixture was then allowed to stand at 105 °C for four h. Upon completion of the reaction as indicated by TLC (ethyl acetate/methyl alcohol, 15:1/*v*:*v*), the mixture was diluted with water (100 mL) and extracted with dichloromethane (30 mL × 3). The combined dichloromethane extracts were dried over anhydrous Na_2_SO_4_, filtered, concentrated in vacuo, and the crude product was purified by column chromatography (silica gel) using ethyl acetate/methanol (10:1/*v*:*v*) as the eluent to afford a pure target compound. Compound **5a** was obtained 0.31 g at a yield of 75%. Compounds **5b**–**5p** were obtained by the same method. Representative data regarding **5a** are listed below. Data for compounds **5b**–**5p** are given in the Supporting Information.

*5,7-Dimethoxy-2-(3,4,5-trimethoxyphenyl)-3-O-(N-(5-((ethyl)thio)-1,3,4-thiadiazol-2-yl) acetamido-2-yl)-4H-chromen-4-one* (**5a**). White solid; yield: 75%; m.p. 223–225 °C; ^1^H-NMR (500 MHz, CDCl_3_) δ: 12.71 (s, 1H, NH), 7.24 (s, 2H, Ar-H), 6.54 (d, *J* = 2.2 Hz, 1H, Ar-H), 6.40 (d, *J* = 2.2 Hz, 1H, Ar-H), 4.48 (s, 2H, O-CH_2_), 3.98 (s, 3H, OCH_3_), 3.96 (s, 3H, OCH_3_), 3.94 (s, 6H, OCH_3_), 3.93 (s, 3H, OCH_3_), 3.27 (q, *J* = 7.4 Hz, 2H, S-CH_2_), 1.44 (t, *J* = 7.4 Hz, 3H, CH_3_); ^13^C-NMR (126 MHz, CDCl_3_) δ: 174.27, 167.50, 164.93, 161.18, 160.43, 158.98, 158.03, 154.18, 153.48, 140.82, 140.77, 124.62, 108.56, 105.82, 96.36, 92.62, 72.53, 61.09, 56.49, 55.98, 28.71, 14.72. ESI-HRMS (*m*/*z*), calcd. for C_26_H_27_N_3_O_9_S_2_ [M + H] ^+^ 590.1262, found 590.1252.

### 3.2. Biological Assays

The antibacterial activities of compounds **5a**–**5p** against *Xac* (Shanghai Jiao Tong University, China), *Rs* (Guizhou University, China), and *Xoo* (Nanjing Agricultural University, China) were evaluated based on the turbidimeter test [25,26,27]. The test compounds were dissolved in 150 μL of dimethyl sulfoxide (DMSO) and diluted with water containing Tween−20 (0.1%) to obtain final concentrations of 100 and 50 μg/mL. Pure DMSO in sterile distilled water served as a blank control, whereas TC and BT served as positive controls. Approximately one mL of liquid sample was added to the nontoxic nutrient broth (NB; three g of beef extract, five g of peptone, one g of yeast powder, 10 g of glucose, and one L of distilled water, Adjust pH = 7.0–7.2 with 10% NaOH) liquid medium in four mL tubes. Then, approximately 40 *μ*L of NB containing bacterium *Xac*/*Rs*/*Xoo* was added to five mL of solvent NB containing the test compounds or TC/BT. The above test tubes were incubated at 30 ± 1 °C with continuous shaking at 180 rpm for 1–3 days. Bacterial growth was monitored with a spectrophotometer by measuring the optical density at 600 nm (OD_600_), given by corrected turbidity values = OD_bacterium_ − OD_no bacterium_. The relative inhibitory rate (*I*%) of the circle mycelium compared with a blank assay was calculated as follows: *I* = (*C*_tur_ − *T*_tur_)/*C*_tur_ × 100%. The *C*_tur_ represents the corrected turbidity value of bacterial growth in untreated NB (blank control), and *T*_tur_ is the corrected turbidity value of bacterial growth in treated NB. Similarly, the excellent antibacterial activities of the target compounds and TC/BT were further assessed against the bacteria *Xac*, *Rs*, and *Xoo* at five double-declining concentrations (100, 50, 25, 12.5, and 6.25 μg/mL) to obtain their EC_50_ values, which were statistically determined using probit analysis within the software package SPSS 17.0. The listed EC_50_ values in Table 2 are the average of three trials.

The curative, protection, and inactivation activities against TMV of the target compounds were tested using previously reported methods [7,29]. The commercial compound RBV was used as a comparison and an average of three replications were conducted for each sample.

## 4. Conclusions

In conclusion, a series of myricetin derivatives containing amide, 1,3,4-oxadiazole, and thioether moieties were developed as potential antibacterial and antiviral agents, and sixteen novel compounds were synthesized. The bioassay results indicated that some of the target compounds possessed excellent antibacterial activities against the plant pathogens *Xoo*, *Rs,* and *Xoo*. For example, compounds **5a**, **5f**, **5g**, **5h**, **5i**, and **5l** showed strong antibacterial activity against *Xac*, with the EC_50_ values of 11.5–27.3 μg/mL, which are significantly better than the commercial bactericides BT (34.7 μg/mL) and TC (41.1% μg/mL). An anti-TMV activity test for the target compounds was performed, and among these compounds, the curative, protection and inactivation activities of **5g** were 49.9%, 52.9%, and 73.3%, respectively, which are better than that of the commercial antiviral RBV (40.6, 51.1, and 71.1%, respectively). It is noteworthy that most of the myricetin derivatives obtained by derivatization had better antibacterial and antiviral activities than those of myricetin. The above studies showed that myricetin derivatives containing amide, 1,3,4-oxadiazole, and thioether moieties could serve as promising candidates for the development of new and more efficient agricultural bactericides against bacterial pathogens and plant viruses.

On the basis of the previous work [18], the hydrophilic amide structure was introduced to improve the water-solubility of target compounds and enrich the structure type and quantity of myricetin derivatives. Extending the linking group between myricetin and the 1,3,4-thiadiazole also made the molecules more flexible and provided some preliminary structure-activity relationships. The bioassays indicated that 1,3,4-thiadiazole and myricetin could not bind to the target protein of *Rs*, *Xoo,* and TMV by extending the linking group between 1,3,4-thiadiazole and myricetin.

## Figures and Tables

**Table 1:** Antibacterial activities of the target compounds (**5a–5p**) against plant pathogenic bacteria in vitro.

Compd.	Inhibition Rate ^a^/%					
	*Xac*		*Rs*		*Xoo*	
	100 μg/mL	50 μg/mL	100 μg/mL	50 μg/mL	100 μg/mL	50 μg/mL
**5a**	75.7 ± 5.3	64.3 ± 3.1	70.4 ± 7.5	50.2 ± 6.5	59.1 ± 2.3	54.4 ± 2.9
**5b**	54.7 ± 1.9	53.5 ± 1.1	41.5 ± 7.5	16.4 ± 0.9	51.7 ± 2.6	44.2 ± 3.4
**5c**	51.3 ± 4.9	18.9 ± 2.6	68.7 ± 6.5	63.4 ± 4.6	62.7 ± 2.8	44.1 ± 6.7
**5d**	39.3 ± 1.4	34.7 ± 1.7	35.3 ± 3.0	37.0 ± 2.0	59.1 ± 2.6	58.0 ± 1.7
**5e**	44.4 ± 2.8	15.2 ± 5.3	78.0 ± 1.5	60.5 ± 3.1	69.8 ± 8.2	62.0 ± 3.0
**5f**	75.0 ± 5.9	60.3 ± 3.6	33.5 ± 9.3	3.0 ± 1.7	68.4 ± 3.5	50.4 ± 2.5
**5g**	89.2 ± 1.9	79.5 ± 1.6	29.7 ± 0.8	20.6 ± 0.7	65.6 ± 5.8	61.4 ± 1.5
**5h**	74.3 ± 2.8	68.3 ± 5.6	41.5 ± 0.3	38.5 ± 2.0	69.5 ± 1.7	33.7 ± 2.8
**5i**	89.5 ± 6.5	68.7 ± 2.7	85.6 ± 4.9	64.8 ± 1.7	78.3 ± 6.9	53.0 ± 1.8
**5j**	41.5 ± 4.1	29.2 ± 3.0	66.3 ± 2.0	59.6 ± 4.9	44.4 ± 5.2	36.9 ± 3.9
**5k**	73.5 ± 6.7	51.6 ± 2.7	28.9 ± 2.1	25.6 ± 1.1	48.3 ± 2.8	47.5 ± 2.5
**5l**	75.3 ± 5.3	55.3 ± 4.1	34.9 ± 4.5	31.2 ± 4.9	54.1 ± 4.0	36.8 ± 1.7
**5m**	72.6 ± 4.8	35.2 ± 1.7	41.4 ± 7.3	34.7 ± 5.8	55.3 ± 2.8	50.2 ± 1.0
**5n**	55.3 ± 2.1	47.9 ± 1.4	52.5 ± 2.9	49.9 ± 4.2	44.8 ± 0.7	43.8 ± 3.4
**5o**	55.6 ± 7.7	46.8 ± 7.0	33.2 ± 2.3	8.3 ± 0.8	77.6 ± 4.3	42.3 ± 1.6
**5p**	67.4 ± 6.8	51.1 ± 7.0	36.7 ± 4.0	35.0 ± 1.3	47.8 ± 7.5	31.8 ± 2.6
MYR ^b^	40.7 ± 4.8	31.1 ± 3.0	57.1 ± 7.5	44.2 ± 3.4	68.5 ± 8.0	53.2 ± 9.4
BT ^c^	73.3 ± 8.2	55.0 ± 4.5	69.7 ± 1.9	57.6 ± 3.1	71.1 ± 3.8	36.5 ± 2.9
TC ^c^	67.7 ± 3.7	44.3 ± 4.0	35.3 ± 3.8	18.9 ± 4.4	36.1 ± 1.3	35.8 ± 2.1

^a^ Average of three replicates. ^b^ The lead compound of myricetin. ^c^ The commercial agricultural bactericides, Bismerthiazol, and Thiodiazole copper were used in a comparison of antibacterial activity.

**Table 2:** EC_50_ values of the title compounds against plant pathogenic bacteria in vitro.

Bacteria	Compd.	R	Toxic Regression Equation	r	EC_50_/(μg/mL)
*Xac*	**5a**	CH_3_	y = 1.2309x + 3.2852	0.9814	24.7 ± 2.7
	**5f**	2-F-Ph	y = 1.1496x+3.3598	0.9978	26.7 ± 1.3
	**5g**	4-F-Ph	y = 1.3129x + 3.5997	0.9928	11.5 ± 1.8
	**5h**	2-CH_3_-Ph	y = 1.3011x + 3.1703	0.9882	25.5 ± 3.2
	**5i**	3-CH_3_-Ph	y = 1.5172x + 3.0628	0.9740	18.9 ± 2.6
	**5l**	4-CF_3_-Ph	y = 1.0060x + 3.5803	0.9869	27.3 ± 2.9
	BT ^a^	-	y = 1.2497x + 3.0744	0.9755	34.7 ± 1.3
	TC ^a^	-	y = 1.1587x + 3.1306	0.9920	41.1 ± 3.7
*Rs*	**5a**	CH_3_	y = 1.0299x + 3.4622	0.9675	31.3 ± 2.5
	**5c**	2-Cl-Ph	y =0.7749x + 3.8771	0.9861	28.1 ± 4.2
	**5e**	2,4-diCl-Ph	y = 1.7574x + 2.1963	0.9954	39.4 ± 3.4
	**5i**	3-CH_3_-Ph	y = 2.0968x + 1.7437	0.9873	35.7 ± 1.6
	BT ^a^	-	y = 1.2725x + 2.6703	0.9839	67.2 ± 2.2
	TC ^a^	-	y = 1.2681x + 2.3203	0.9927	112.5 ± 4.7
*Xoo*	**5e**	2,4-diCl-Ph	y = 1.3410x + 2.9457	0.9951	34.0 ± 3.2
	**5f**	2-F-Ph	y = 1.3885x + 2.7900	0.9839	44.9 ± 3.5
	**5h**	2-CH_3_-Ph	y = 2.0971x + 1.1978	0.9918	65.0 ± 2.1
	**5i**	3-CH_3_-Ph	y = 1.3586x + 2.9710	0.9891	31.2 ± 2.4
	**5o**	4-CF_3_-Ph	y = 1.8004x + 1.9332	0.9792	50.5 ± 5.7
	BT ^a^	-	y = 1.6145x + 1.7430	0.9887	45.3 ± 3.0
	TC ^a^	-	y = 1.8721x + 1.8993	0.9830	105.1 ± 4.6

^a^ The commercial agricultural antibacterial agents Thiodiazole copper (TC) and Bismerthiazol (BT) were used as control agents.

**Table 3:** Antiviral activities of the test compounds against TMV in vivo at 500 μg/mL.

Compd.	R	Inhibition Rate ^a^/%		
		Curative	Protection	Inactivation
**5a**	CH_3_	38.0 ± 6.3	40.0 ± 6.9	53.6 ± 5.4
**5b**	Ph	26.0 ± 4.9	36.0 ± 6.0	71.5 ± 5.1
**5c**	2-Cl-Ph	27.1 ± 4.1	40.8 ± 7.1	72.9 ± 5.1
**5d**	4-Cl-Ph	35.6 ± 4.9	42.2 ± 7.3	62.0 ± 2.8
**5e**	2,4-diCl-Ph	22.3 ± 1.8	43.2 ± 4.6	76.7 ± 3.5
**5f**	2-F-Ph	42.2 ± 8.9	49.2 ± 8.4	61.2 ± 5.9
**5g**	4-F-Ph	49.9 ± 5.0	52.9 ± 5.2	73.3 ± 2.8
**5h**	2-CH_3_-Ph	31.0 ± 6.4	34.7 ± 5.7	58.1 ± 4.6
**5i**	3-CH_3_-Ph	44.2 ± 5.3	45.3 ± 7.2	57.0 ± 4.6
**5j**	4-CH_3_O-Ph	30.6 ± 1.0	54.5 ± 5.7	63.5 ± 4.9
**5k**	3-CF_3_-Ph	39.9 ± 4.2	46.4 ± 5.9	60.2 ± 3.9
**5l**	4-CF_3_-Ph	45.3 ± 4.1	49.9 ± 7.3	59.5 ± 6.2
**5m**	3-pyridyl	33.2 ± 4.8	32.2 ± 8.8	52.5 ± 5.5
**5n**	4-pyridyl	43.1 ± 3.3	36.1 ± 8.6	62.6 ± 3.3
**5o**	2-Cl-5-thiazolyl	33.8 ± 7.6	44.8 ± 5.9	65.3 ± 2.7
**5p**	4-NO_2_-Ph	35.9 ± 3.7	47.7 ± 2.4	64.6 ± 4.1
MYR ^b^	-	36.7 ± 6.3	42.3 ± 6.5	51.5 ± 3.7
RBV ^c^	-	40.6 ± 2.5	51.1 ±2.3	71.1 ± 4.2

^a^ Average of three replicates. ^b^ The lead compound of myricetin(MYR). ^c^ The commercial antiviral agent Ribavirin (RBV).

## Supplementary Materials

The supplementary materials containing the yield relationship, ^1^H-NMR, ^13^C-NMR, HRMS dates and spectra of the title compounds **5a**–**5p** are available online.
