# Supplementary material for: Design, Synthesis, and Biological Activity of Novel Myricetin Derivatives Containing Amide, Thioether, and 1,3,4-Thiadiazole Moieties
Source: Molecules. 2018 Nov 29;23(12):3132. doi: 10.3390/molecules23123132 (PMC6321191; doi:10.3390/molecules23123132)
Supplement: Supplementary file 1 [file molecules-23-03132-s001.pdf]

Supplementary Materials

Design, synthesis, biological activity study of novel myricetin derivatives containing the amide, thioether and 1,3,4-thiadiazole moiety

Xianghui Ruan†, Cheng Zhang†, Shichun Jiang, Tao Guo, Rongjiao Xia, Ying Chen, Xu Tang, Wei Xue*

Key Laboratory of Green Pesticide and Agriculture Bioengineering, Ministry of Education,
Guizhou University, Huaxi District, Guiyang 550025, China; E-Mails: rxh9105@163.com (X.R.);

† These authors contributed equally to this work.

***** Corresponding author: Wei Xue; e-mail: wxue@gzu.edu.cn (W.X.); Tel/Fax: 0086-851-88292090

Content

1. **The yield relationship of compounds 5a-5p** 2

2. **The data of title compounds 5a–5p** 2

3. **Spectrogram of title compounds 5a-5p** 8

1. The yield relationship of compounds 5a-5p

Table 1 The yield relationship of compounds **5a-5p**

Compound	m(g)	Yeid(%)	Compound	m(g)	Yeid(%)
**5a**	0.31	75	**5i**	0.37	77
**5b**	0.31	66	**5j**	0.27	61
**5c**	0.36	73	**5k**	0.27	53
**5d**	0.36	76	**5l**	0.25	50
**5e**	0.29	58	**5m**	0.22	48
**5f**	0.28	59	**5n**	0.23	49
**5g**	0.29	62	**5o**	0.22	44
**5h**	0.35	69	**5p**	0.17	34

2. The data of title compounds 5a–5p

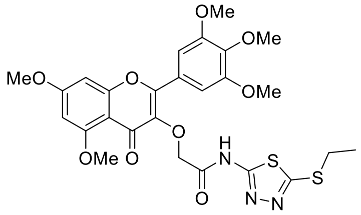

Data for *5,7-Dimethoxy-2-(3,4,5-trimethoxyphenyl)-3-O-(N-(5-((ethyl)thio)-1,3,4-thiadiazol-2-yl)acetamido-2-yl)-4H-chromen-4-one (**5a**)* White solid; yield: 75%; m.p. 223-225 °C; ^{1}H NMR (500 MHz, $CDCl_3$) *δ*: 12.71 (s, 1H, NH), 7.24 (s, 2H, Ar-H), 6.54 (d, *J* = 2.2 Hz, 1H, Ar-H), 6.40 (d, *J* = 2.2 Hz, 1H, Ar-H), 4.48 (s, 2H, O-CH_2), 3.98 (s, 3H, OCH_3), 3.96 (s, 3H, OCH_3), 3.94 (s, 6H, OCH_3), 3.93 (s, 3H, OCH_3), 3.27 (q, *J* = 7.4 Hz, 2H, S-CH_2), 1.44 (t, *J* = 7.4 Hz, 3H, CH_3); ^{13}C NMR (126 MHz, $CDCl_3$) *δ*: 174.27, 167.50, 164.93, 161.18, 160.43, 158.98, 158.03, 154.18, 153.48, 140.82, 140.77, 124.62, 108.56, 105.82, 96.36, 92.62, 72.53, 61.09, 56.49, 55.98, 28.71, 14.72. ESI-HRMS (m/z), calcd for $C_{26}H_{27}N_3O_9S_2$ $[M+H]^+$ 590.1262, found 590.1252.

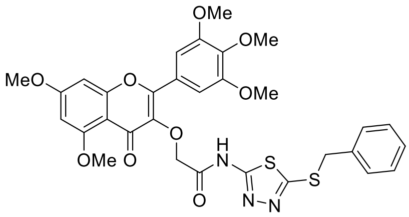

Data for *5,7-Dimethoxy-2-(3,4,5-trimethoxyphenyl)-3-O-(N-(5-((phenyl)thio)-1,3,4-thiadiazol-2-yl)acetamido-2-yl)-4H-chromen-4-one (**5b**)* Brown solid; yield: 66%; m.p. 140-142 °C; ^{1}H NMR (500 MHz, $CDCl_3$) *δ*: 12.71 (s, 1H, NH), 7.40 (d, *J* = 6.4 Hz, 2H, Ph-H), 7.34-7.29 (m, 3H, Ph-H), 7.24 (s, 2H, Ar-H), 6.54 (s, 1H, Ar-H), 6.40 (s, 1H, Ar-H), 4.49 (s, 2H, O-CH_2), 4.47 (s, 2H, S-CH_2), 3.98 (s, 3H, OCH_3), 3.95 (s, 3H, OCH_3), 3.94 (s, 9H, 3×OCH_3); ^{13}C NMR (126 MHz, $CDCl_3$) *δ*: 174.28, 167.48, 164.97, 161.25, 159.70, 159.02, 158.33, 154.23, 153.53, 140.97, 149.79, 136.23, 129.17, 128.71, 127.78, 124.63, 108.63, 105.96, 96.39, 92.67, 72.55, 61.09, 56.54, 55.97, 38.57. ESI-HRMS (m/z), calcd for $C_{31}H_{29}N_3O_9S_2$ $[M+H]^+$ 652.1418, found 652.1411.

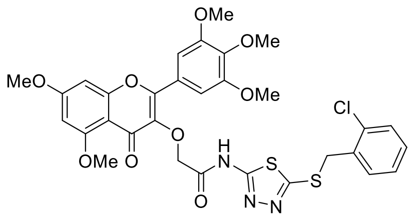

Data for *5,7-Dimethoxy-2-(3,4,5-trimethoxyphenyl)-3-O-(N-(5-((2-chlorobenzyl)thio)-1,3,4-thiadiazol-2-yl)acetamido-2-yl)-4H-chromen-4-one* ***(5c)*** White solid; yield: 73%; m.p. 232-234 °C; ^{1}H NMR (500 MHz, $CDCl_3$) δ: 12.68 (s, 1H, NH), 7.49 (d, J = 8.9 Hz, 1H, Ph-H), 7.38 (d, J = 7.9 Hz, 1H, Ph-H), 7.23 (s, 2H, Ph-H), 7.20 (dd, J = 8.8, 6.8 Hz, 2H, Ph-H), 6.54 (d, J = 2.0 Hz, 1H, Ar-H), 6.41 (d, J = 2.0 Hz, 1H, Ar-H), 4.61 (s, 2H, O-CH_2), 4.46 (s, 2H, S-CH_2), 3.99 (s, 3H, OCH_3), 3.95 (s, 3H, OCH_3), 3.93 (s, 9H, 3×OCH_3); ^{13}C NMR (126 MHz, $CDCl_3$) δ: 174.34, 167.57, 164.98, 161.22, 159.02, 158.97, 158.67, 154.29, 153.52, 140.95, 140.83, 134.97, 134.30, 133.17, 132.17, 129.54, 127.27, 124.60, 108.59, 105.93, 96.41, 92.66, 72.58, 61.09, 56.52, 55.97, 35.25. ESI-HRMS (m/z), calcd for $C_{31}H_{28}ClN_3O_9S_2$ $[M+H]^+$ 686.1028, found 686.1023.

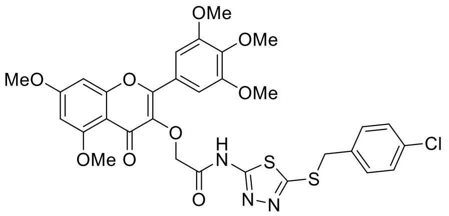

Data for *5,7-Dimethoxy-2-(3,4,5-trimethoxyphenyl)-3-O-(N-(5-((4-chlorobenzyl)thio)-1,3,4-thiadiazol-2-yl)acetamido-2-yl)-4H-chromen-4-one* ***(5d)*** Brown solid; yield: 76%; m.p. 191-193 °C; ^{1}H NMR (500 MHz, DMSO-*d6*) δ: 12.90 (s, 1H, NH), 7.43 (s, 4H, Ph-H), 7.40-7.38 (m, 2H, Ar-H), 6.90 (d, J = 2.2 Hz, 1H, Ar-H), 6.54 (d, J = 2.2 Hz, 1H, Ar-H), 4.82 (s, 2H, O-CH_2), 4.49 (d, J = 3.8 Hz, 2H, S-CH_2), 3.92 (s, 3H, OCH_3), 3.85 (d, J = 6.4 Hz, 9H, 3×OCH_3), 3.73 (s, 3H, OCH_3); ^{13}C NMR (126 MHz, DMSO-*d6*) δ: 172.84, 168.02, 164.61, 160.86, 159.06, 158.73, 158.50, 153.26, 152.13, 140.17, 140.00, 136.58, 132.69, 131.40, 129.03, 125.61, 108.67, 107.32, 106.59, 96.70, 93.76, 70.62, 60.69, 56.73, 56.66, 37.20. ESI-HRMS (m/z), calcd for $C_{31}H_{28}ClN_3O_9S_2$ $[M+H]^+$ 686.1028, found 686.1020.

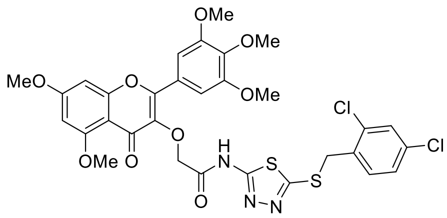

Data for *5,7-Dimethoxy-2-(3,4,5-trimethoxyphenyl)-3-O-(N-(5-((2,4-dichlorobenzyl)thio)-1,3,4-thiadiazol-2-yl)acetamido-2-yl)-4H-chromen-4-one* ***(5e)*** White solid; yield: 58%; m.p. 245-247 °C; ^{1}H NMR (500 MHz, $CDCl_3$) δ: 12.71 (s, 1H, NH), 7.49 (dd, J = 7.2, 2.0 Hz, 1H, Ph-H), 7.38 (dd, J = 7.6, 1.6 Hz, 1H, Ph-H), 7.24 (s, 2H, Ar-H), 7.21 – 7.19 (m, 1H, Ph-H), 6.54 (d, J = 2.2 Hz, 1H, Ar-H), 6.41 (d, J = 2.2 Hz, 1H, Ar-H), 4.61 (s, 2H, O-CH_2), 4.47 (s, 2H, S-CH_2), 3.99 (s, 3H, OCH_3), 3.96 (s, 3H, OCH_3), 3.94 (s, 6H, 2×OCH_3), 3.93 (s, 3H, OCH_3); ^{13}C NMR (126 MHz, $CDCl_3$) δ: 174.37, 167.60, 165.01, 161.30, 159.45, 159.10, 158.52, 154.27, 153.57, 140.87, 134.45, 134.40, 131.47, 129.82, 129.32, 127.08, 124.73, 108.70, 105.90, 96.47, 92.70, 72.60, 61.19, 56.58, 56.06, 36.14. ESI-HRMS (m/z), calcd for $C_{31}H_{27}Cl_2N_3O_9S_2$ $[M+H]^+$ 720.0639, found 720.0640.

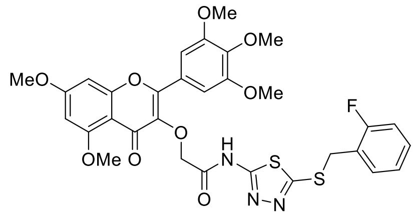

Data for *5,7-Dimethoxy-2-(3,4,5-trimethoxyphenyl)-3-O-(N-(5-((2-fluorophenyl)thio)-1,3,4-thiadiazol-2-yl)acetamido-2-yl)-4H-chromen-4-one* ***(5f)*** White solid; yield: 59%; m.p. 256-258 °C; ^{1}H NMR (500 MHz, $CDCl_3$) δ: 12.75 (s, 1H, NH), 7.42 (t, J = 7.5 Hz, 1H, Ph-H), 7.23 (s, 2H, Ar-H), 7.09-7.01 (m, 3H, Ph-H), 6.53 (d, J = 1.7 Hz, 1H, Ar-H), 6.39 (d, J = 1.9 Hz, 1H, Ar-H), 4.51 (s, 2H, O-CH_2), 4.46 (s, 2H, S-CH_2), 3.97 (s, 3H, OCH_3), 3.95 (s, 3H, OCH_3), 3.93 (s, 6H, 2×OCH_3), 3.92 (s, 3H, OCH_3); ^{13}C NMR (126 MHz, $CDCl_3$) δ: 174.35, 167.58, 165.03, 161.31, 161.04 (d, $^1J_{CF}$ = 248.2 Hz), 159.33, 159.08, 158.60, 154.28, 153.60, 141.03, 140.86, 131.42 (d, $^3J_{CF}$ = 2.6 Hz), 129.73 (d, $^3J_{FC}$ = 8.2 Hz), 124.69, 124.32 (d, $^4J_{FC}$ = 3.3 Hz), 123.85 (d, $^2J_{FC}$ = 21.4 Hz), 115.63 (d, $^2J_{FC}$ = 21.1 Hz), 108.68, 106.03, 96.46, 92.74, 72.62, 61.15, 56.61, 56.56, 56.04, 31.71 (d, $^3J_{FC}$= 2.8 Hz). ESI-HRMS (m/z), calcd for $C_{31}H_{28}FN_3O_9S_2$ $[M+H]^+$ 700.1324, found 700.1314.

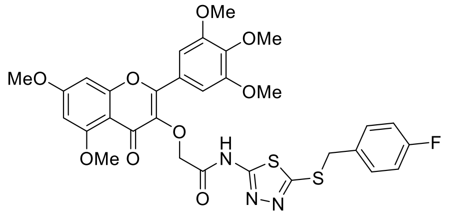

Data for *5,7-Dimethoxy-2-(3,4,5-trimethoxyphenyl)-3-O-(N-(5-((4-fluorophenyl)thio)-1,3,4-thiadiazol-2-yl) acetamido-2-yl)-4H-chromen-4-one* ***(5g)*** White solid; yield: 62%; m.p. 273-274 °C; ^{1}H NMR (500 MHz, $CDCl_3$) δ: 12.70 (s, 1H, NH), 7.39-7.36 (m, 2H, Ph-H), 7.24 (s, 2H, Ar-H), 7.00 (t, J = 8.3 Hz, 2H, Ph-H), 6.54 (s, 1H, Ar-H), 6.40 (s, 1H, Ar-H), 4.48 (s, 2H, O-CH_2), 4.46 (s, 2H, S-CH_2), 3.98 (s, 3H, OCH_3), 3.96 (s, 3H, OCH_3), 3.94 (s, 6H, 2×OCH_3), 3.93 (s, 3H, OCH_3); ^{13}C NMR (126 MHz, $CDCl_3$) δ: 174.2, 167.52, 164.91, 162.22 (d, $^1J_{FC}$= 256.2 Hz), 161.20, 159.26, 159.00, 158.32, 154.16, 153.48, 140.79(d, $^4J_{FC}$= 8.2 Hz), 130.84 (d, $^3J_{FC}$ = 8.3 Hz), 124.62, 115.57 (d, $^2J_{FC}$= 21.6 Hz), 105.83, 96.36, 92.61, 72.48, 61.08, 56.49, 55.96, 37.64. ESI-HRMS (m/z), calcd for $C_{31}H_{28}FN_3O_9S_2$ $[M+H]^+$ 700.1324, found 700.1309.

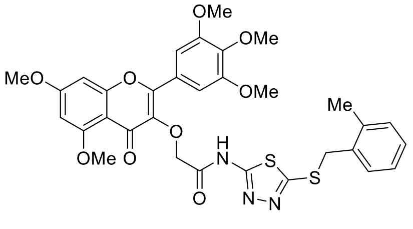

Data for *5,7-Dimethoxy-2-(3,4,5-trimethoxyphenyl)-3-O-(N-(5-((2-methylphenyl)thio)-1,3,4-thiadiazol-2-yl)acetamido-2-yl)-4H-chromen-4-one* ***(5h)*** White solid; yield: 69%; m.p. 268-270 °C; ^{1}H NMR (500 MHz, $CDCl_3$) δ: 12.67 (s, 1H, NH), 7.29 (s, 1H, Ph-H), 7.27 (s, 1H, Ph-H), 7.24 (s, 2H, Ar-H), 7.13 (s, 1H, Ph-H), 7.11 (s, 1H, Ph-H), 6.54 (d, J = 2.2 Hz, 1H, Ar-H), 6.41 (d, J = 2.2 Hz, 1H, Ar-H), 4.47 (s, 2H, O-CH_2), 4.46 (s, 2H, S-CH_2), 3.99 (s, 3H, OCH_3), 3.96 (s, 3H, OCH_3), 3.94 (s, 6H, OCH_3), 3.93 (s, 3H, OCH_3), 2.33 (s, 3H, CH_3). ^{13}C NMR (126 MHz, $CDCl_3$) δ: 174.22, 167.47, 164.90, 161.20, 159.92, 158.99, 158.23, 154.15, 153.48, 140.83, 140.73, 137.54, 133.03, 129.52, 129.38, 129.05, 128.93, 124.65, 108.61,

105.85, 96.35, 92.62, 72.45, 61.08, 56.49, 55.95, 38.35, 21.16. ESI-HRMS (m/z), calcd for $C_{32}H_{31}N_3O_9S_2$ $[M+H]^+$ 666.1575, found 666.1564.

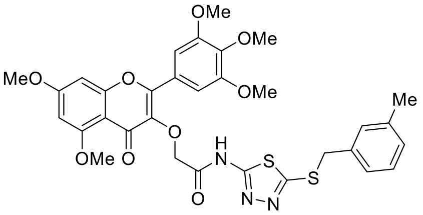

Data for

*5,7-Dimethoxy-2-(3,4,5-trimethoxyphenyl)-3-O-(N-(5-((3-methylbenzyl)thio)-1,3,4-thiadiazol-2-yl)acetamido-2-yl)-4H-chromen-4-one (**5i**)* White solid; yield: 77%; m.p. 247-249 °C; ^{1}H NMR (500 MHz, $CDCl_3$) δ: 12.68 (s, 1H, NH), 7.24 (s, 2H, Ar-H), 7.21 (s, 1H, Ph-H), 7.20 (s, 1H, Ph-H), 7.18 (s, 1H, Ph-H), 7.07 (d, J = 6.1 Hz, 1H, Ph-H), 6.53 (d, J = 2.2 Hz, 1H, Ar-H), 6.39 (d, J = 2.1 Hz, 1H, Ar-H), 4.48 (s, 2H, O-CH_2), 4.45 (s, 2H, S-CH_2), 3.97 (s, 3H, OCH_3), 3.95 (s, 3H, OCH_3), 3.93 (s, 6H, 2×OCH_3), 3.92 (s, 3H, OCH_3), 2.33 (s, 3H, CH_3); ^{13}C NMR (126 MHz, $CDCl_3$) δ: 174.22, 167.48, 164.92, 161.22, 159.93, 159.00, 158.25, 154.16, 153.50, 140.90, 140.74, 138.42, 135.98, 129.84, 128.58, 126.20, 124.65, 108.63, 105.93, 96.37, 92.65, 72.46, 61.08, 56.52, 55.96, 38.52, 21.34. ESI-HRMS (m/z), calcd for $C_{32}H_{31}N_3O_9S_2$ $[M+H]^+$ 666.1575, found 666.1565.

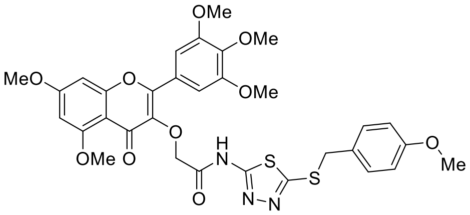

Data for

*5,7-Dimethoxy-2-(3,4,5-trimethoxyphenyl)-3-O-(N-(5-((4-methoxyphenyl)thio)-1,3,4-thiadiazol-2-yl)acetamido-2-yl)-4H-chromen-4-one (**5j**)* White solid; yield: 61%; m.p. 271-272 °C; ^{1}H NMR (500 MHz, $CDCl_3$) δ: 12.74 (s, 1H, NH), 7.33 – 7.30 (m, 2H, Ph-H), 7.24 (s, 2H, Ar-H), 6.86 – 6.82 (m, 2H, Ph-H), 6.54 (d, J = 2.2 Hz, 1H, Ar-H), 6.40 (d, J = 2.2 Hz, 1H, Ar-H), 4.47 (s, 2H, O-CH_2), 4.44 (s, 2H, S-CH_2), 3.98 (s, 3H, OCH_3), 3.96 (s, 3H, OCH_3), 3.94 (s, 6H, 2×OCH_3), 3.93 (s, 3H, OCH_3), 3.79 (s, 3H, OCH_3). ^{13}C NMR (126 MHz, $CDCl_3$) δ: 174.26, 167.47, 164.92, 161.17, 159.85, 159.15, 158.97, 158.23, 154.17, 153.47, 140.81, 140.75, 130.37, 128.08, 124.62, 114.08, 108.55, 105.82, 96.36, 92.62, 72.51, 61.08, 56.49, 55.97, 55.27, 38.14. ESI-HRMS (m/z), calcd for $C_{32}H_{31}N_3O_{10}S_2$ $[M+H]^+$ 682.1524, found 682.1508.

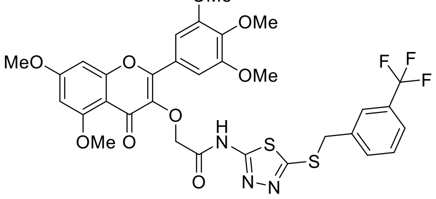

Data for

*5,7-Dimethoxy-2-(3,4,5-trimethoxyphenyl)-3-O-(N-(5-((3-trifluoromethylphenyl)thio)-1,3,4-thiadiazol-2-yl)acetamido-2-yl)-4H-chromen-4-one (**5k**)* White solid; yield: 53%; m.p. 184-185 °C; ^{1}H NMR (500 MHz, $(CD_3)_2CO$) δ: 7.86-7.75 (m, 3H), 7.62 (d, J = 7.6 Hz, 1H), 7.58 (d, J = 7.7 Hz, 1H), 7.44 (s, 1H), 7.38 (s, 2H), 6.66 (dd, J = 124.3, 2.2 Hz, 1H), 5.17 (s, 2H), 4.63 (s, 2H), 3.96-3.81 (m, 15H). ^{13}C NMR (126 MHz, $(CD_3)_2CO$) δ: 167.22, 167.08, 166.05, 165.87, 162.10,159.88, 159.32, 159.18, 158.95, 154.56, 154.26,

144.04, 141.83, 139.72, 133.90, 130.98, 130.69, 130.34, 126.67, 126.26, 125.98, 125.15, 125.06, 124.11, 108.20, 107.19, 96.97, 93.90, 63.51, 60.73, 56.81, 56.64, 37.83. ESI-HRMS (m/z), calcd for $C_{32}H_{28}F_3N_3O_9S_2$ $[M+H]^+$ 720.1292, found 720.1281.

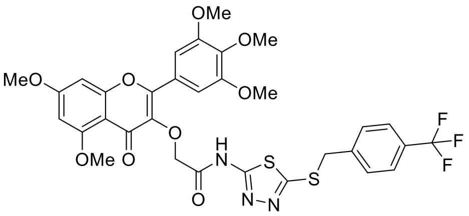

Data for *5,7-Dimethoxy-2-(3,4,5-trimethoxyphenyl)-3-O-(N-(5-((4-trifluoromethylphenyl)thio)-1,3,4-thiadiazol-2-yl)acetamido-2-yl)-4H-chromen-4-one* ***(5l)*** White solid; yield: 50%; m.p. 191-193 °C; ^{1}H NMR (500 MHz, $(CD_3)_2CO$) δ: 7.85 – 7.76 (m, 2H, Ph-H), 7.64-7.56 (m, 2H, Ph-H), 7.38 (s, 2H, Ar-H), 6.79 (d, J = 2.2 Hz, 1H, Ar-H), 6.54 (d, J = 2.2 Hz, 1H, Ar-H), 5.17 (s, 2H, O-CH_2), 4.63 (s, 2H, S-CH_2), 3.93-3.82 (m, 15H, 5×OCH_3). ^{13}C NMR (126 MHz, $(CD_3)_2CO$) δ: 167.08, 166.05,165.88, 162.11, 159.89, 159.32, 159.19, 154.57, 154.26, 144.05, 141.83, 139.73, 133.91, 130.99, 130.35, 126.68, 125.13, 108.21, 107.20, 96.97, 93.91, 63.51, 60.74, 56.65, 56.61, 37.84. ESI-HRMS (m/z), calcd for $C_{32}H_{28}F_3N_3O_9S_2$ $[M+H]^+$ 720.1292, found 720.1281.

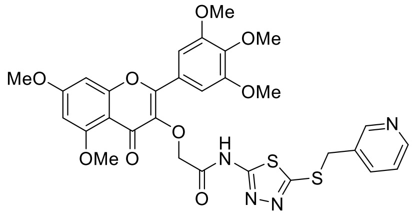

Data for *5,7-Dimethoxy-2-(3,4,5-trimethoxyphenyl)-3-O-(N-(5-((3-pyridyl)thio)-1,3,4-thiadiazol-2-yl)acetamido-2-yl)-4H-chromen-4-one* ***(5m)*** White solid; yield: 48%; m.p. 252-254 °C; ^{1}H NMR (500 MHz, $CDCl_3$) δ: 12.86 (s, 1H, NH), 8.62 (s, 1H, pyridine-H), 8.50 (d, J = 3.9 Hz, 1H, pyridine-H), 7.77 (dt, J = 7.8, 1.8 Hz, 1H, pyridine-H), 7.25 (dd, J = 7.6, 2.7 Hz, 1H, pyridine-H), 7.23 (s, 2H, Ar-H), 6.53 (d, J = 2.2 Hz, 1H, Ar-H), 6.39 (d, J = 2.2 Hz, 1H, Ar-H), 4.47 (s, 2H, O-CH_2), 4.46 (s, 2H, S-CH_2), 3.96 (s, 3H, OCH_3), 3.94 (s, 3H, OCH_3), 3.93 (s, 6H, 2×OCH_3), 3.92 (s, 3H, OCH_3). ^{13}C NMR (126 MHz, $CDCl_3$) δ: 174.32, 167.59, 164.95, 161.19, 158.99, 158.99, 158.61, 154.22, 153.48, 150.18, 148.99, 140.84, 140.81, 136.75, 132.57, 124.59, 123.52, 108.54, 105.83, 96.38, 92.62, 72.55, 61.08, 56.50, 55.98, 35.21. ESI-HRMS (m/z), calcd for $C_{30}H_{28}N_4O_9S_2$ $[M+H]^+$ 653.1371, found 653.1366.

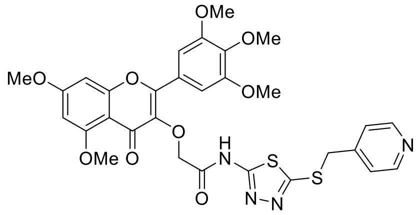

Data for *5,7-Dimethoxy-2-(3,4,5-trimethoxyphenyl)-3-O-(N-(5-((4-pyridyl)thio)-1,3,4-thiadiazol-2-yl)acetamido-2-yl)-4H-chromen-4-one* ***(5n)*** White solid; yield: 49%; m.p. 254-256 °C; ^{1}H NMR (500 MHz, $CDCl_3$) δ: 12.88 (s, 1H, NH), 8.55 (dd, J = 4.5, 1.6 Hz, 2H, pyridine-H), 7.34 (dd, J = 4.5, 1.5 Hz, 2H, pyridine-H), 7.24 (s, 2H, Ar-H), 6.54 (d, J = 2.2 Hz, 1H, Ar-H), 6.40 (d, J = 2.2 Hz, 1H, Ar-H), 4.47 (s, 2H, O-CH_2), 4.45 (s, 2H, S-CH_2), 3.98 (s, 3H, OCH_3), 3.96 (s, 3H, OCH_3), 3.94 (s, 6H, 2×OCH_3), 3.93 (s, 3H, OCH_3). ^{13}C NMR (126 MHz, $CDCl_3$)

δ: 174.33, 167.61, 164.95, 161.19, 158.99, 158.58, 158.35, 154.23, 153.49, 150.08, 145.66, 140.87, 140.82, 124.58, 123.94, 108.55, 105.85, 96.38, 92.63, 72.56, 61.08, 56.50, 55.97, 36.67. ESI-HRMS (m/z), calcd for $C_{30}H_{28}N_4O_9S_2$ $[M+H]^+$ 653.1371, found 653.1361.

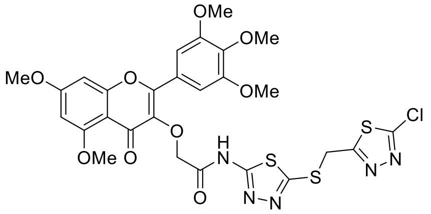

Data for

5,7-Dimethoxy-2-(3,4,5-trimethoxyphenyl)-3-O-(N-(5-((2-chlorothiazolyl)thio)-1,3,4-thiadiazol-2-yl)acetamido-2-yl)-4H-chromen-4-one ***(5o)*** White solid; yield: 44%; m.p. 231-233 °C; 6.90 (d, *J* = 2.1 Hz, 1H, Ar-H), 6.53 (d, *J* = 2.2 Hz, 1H, Ar-H), 4.83 (s, 2H, O-CH_2), 4.73 (s, 2H, S-CH_2), 3.92 (s, 3H, OCH_3), 3.86 (s, 9H, 3×OCH_3), 3.73 (s, 3H, OCH_3). ^{13}C NMR (126 MHz, DMSO-*d6*) δ: 172.86, 168.27, 164.60, 160.87, 159.81, 158.73, 157.54, 153.27, 152.09, 150.82, 141.35, 140.19, 140.04, 138.94, 125.63, 108.69, 106.63, 96.70, 93.76, 70.71, 60.69, 56.74, 56.68, 29.85. ESI-HRMS (m/z), calcd for $C_{28}H_{25}ClN_4O_9S_3$ $[M+H]^+$ 693.0545, found 693.0539 ($[M+H]^+$).

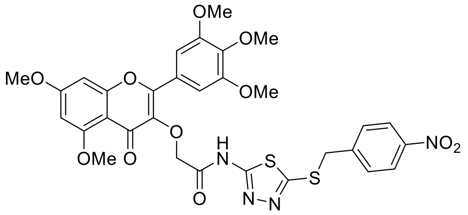

Data for

5,7-Dimethoxy-2-(3,4,5-trimethoxyphenyl)-3-O-(N-(5-((4-nitrobenzyl)thio)-1,3,4-thiadiazol-2-yl)acetamido-2-yl)-4H-chromen-4-one ***(5p)*** White solid; yield: 34%; m.p. 245-247 °C; ^{1}H NMR (500 MHz, DMSO-*d6*) δ: 12.92 (s, 1H, NH), 8.18 (d, *J* = 8.7 Hz, 2H, Ph-H), 7.68 (d, *J* = 8.6 Hz, 2H, Ph-H), 7.43 (s, 2H, Ar-H), 6.89 (d, *J* = 1.7 Hz, 1H, Ar-H), 6.53 (d, *J* = 1.9 Hz, 1H, Ar-H), 4.83 (s, 2H, O-CH_2), 4.63 (s, 2H, S-CH_2), 3.92 (s, 3H, OCH_3), 3.86 (s, 9H, 3×OCH_3), 3.73 (s, 3H, OCH_3). ^{13}C NMR (126 MHz, DMSO-*d6*) δ: 173.72, 171.29, 170.96, 164.98, 160.59, 158.96, 157.86, 156.14, 152.02, 148.36, 145.83, 142.13, 137.49, 130.05, 128.19, 125.26, 108.65, 107.26, 96.91, 94.80, 71.39, 61.77, 57.91, 57.16, 40.62. ESI-HRMS (m/z), calcd for $C_{31}H_{28}N_4O_{11}S_2$ $[M+H]^+$ 653.1371, found 653.1366 ($[M+H]^+$).

3. Spectrogram of title compounds 5a-5p

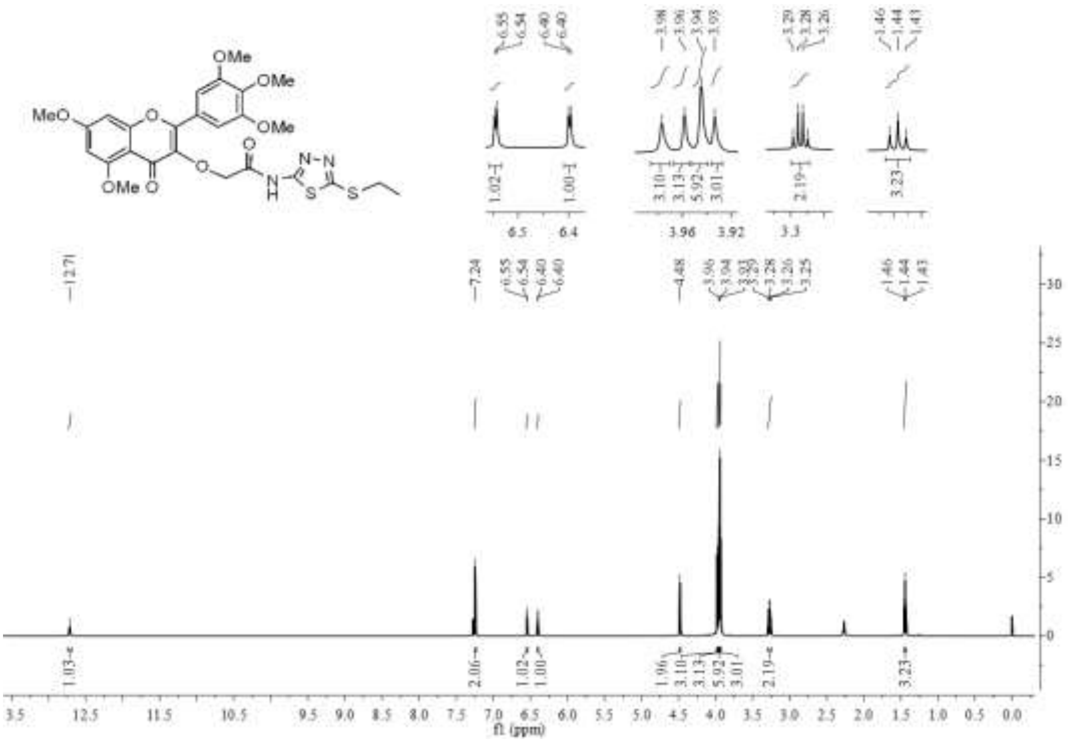

^{1}H NMR of compound **5a**

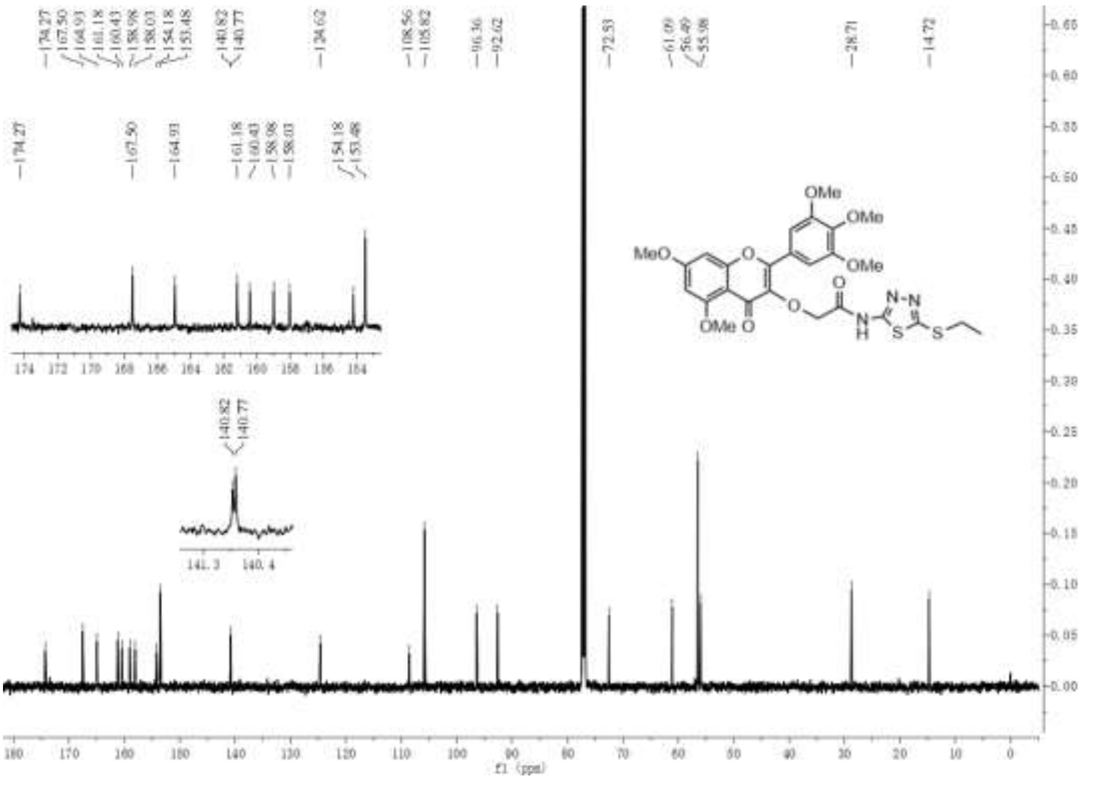

^{13}C NMR of compound **5a**

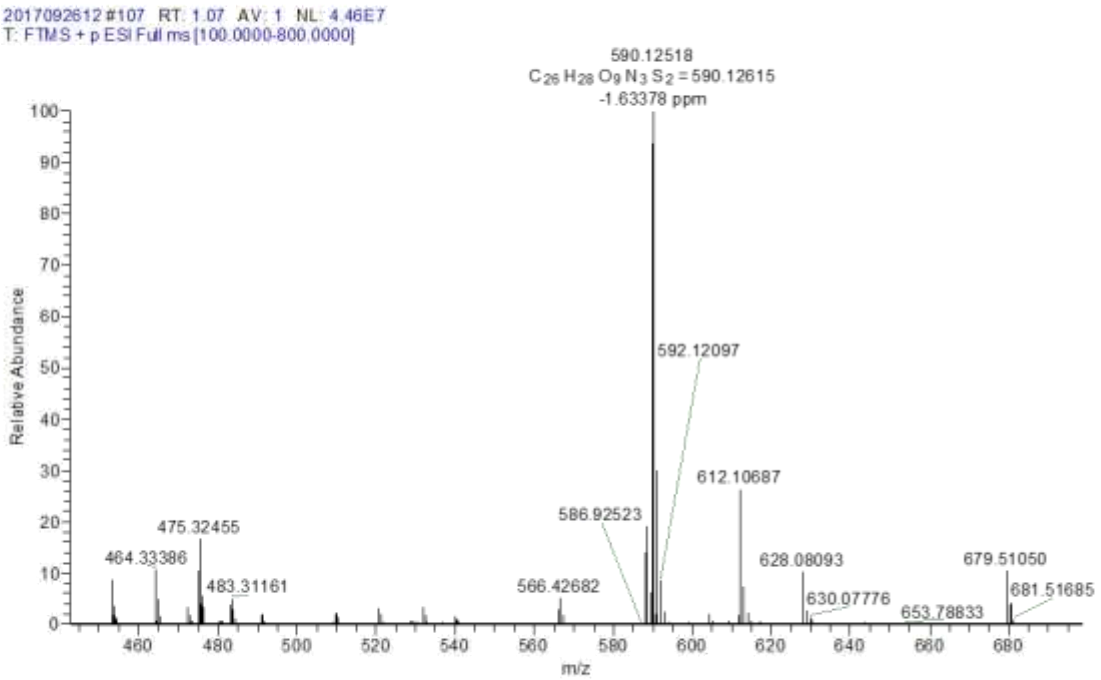

HRMS of compound **5a**

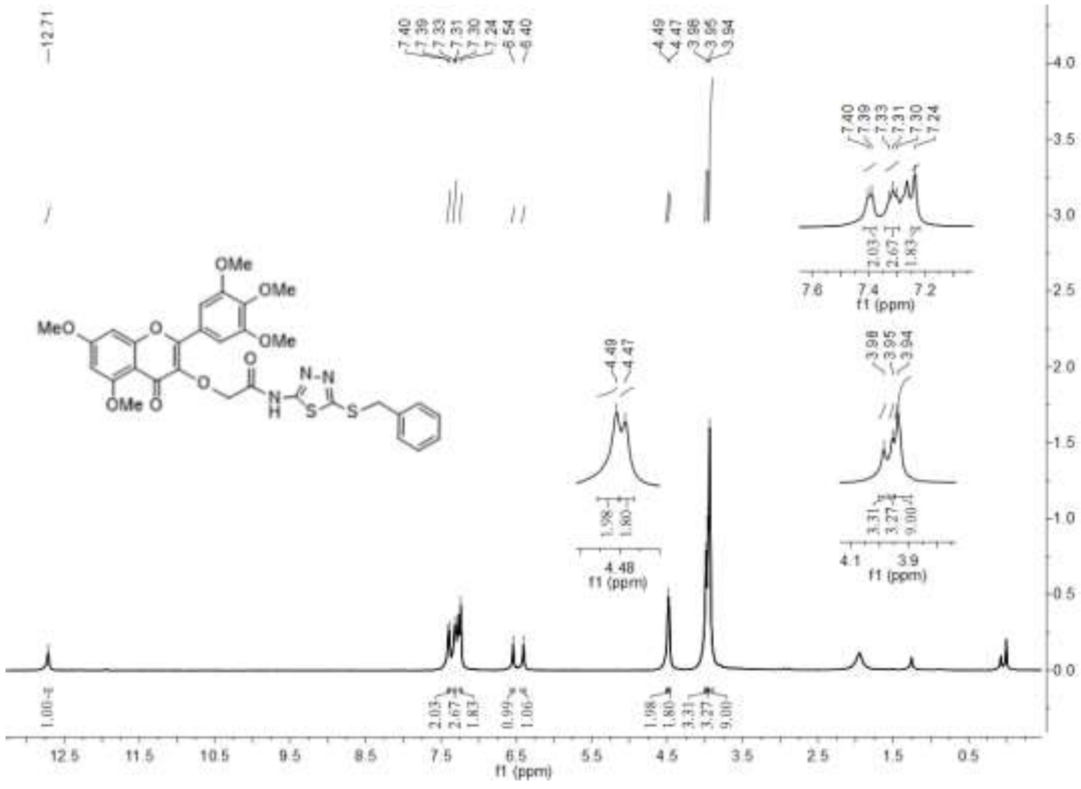

^{1}H NMR of compound **5b**

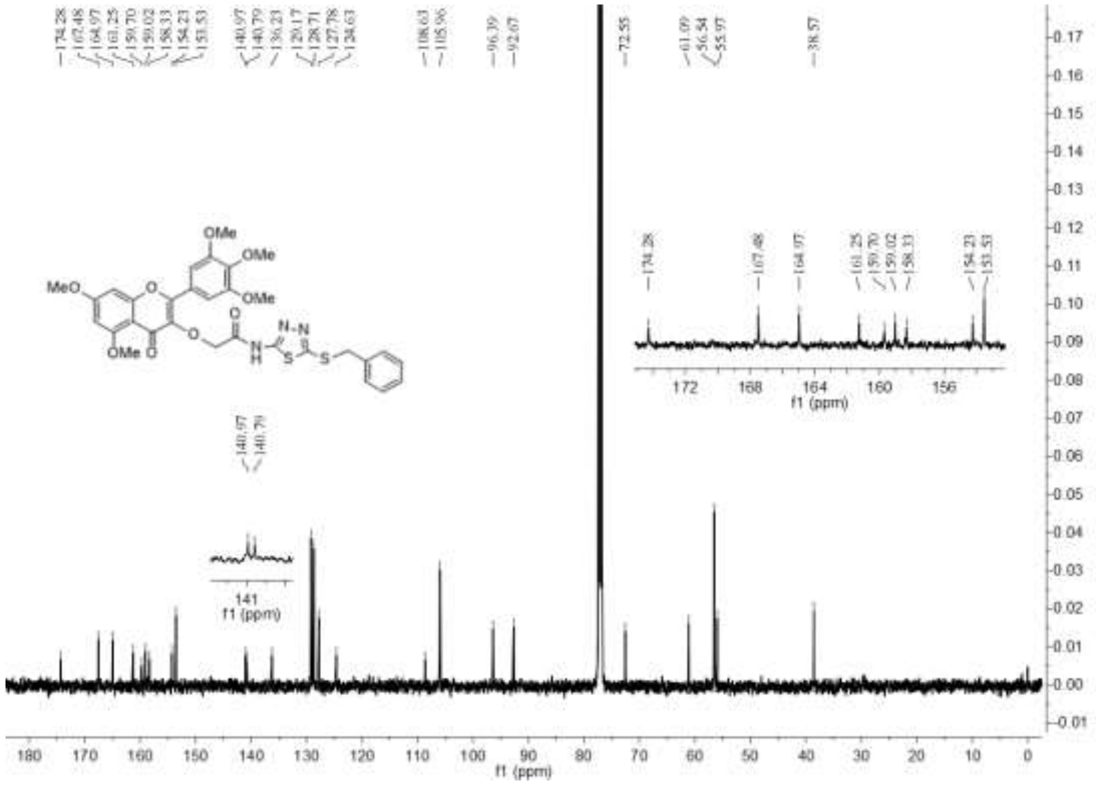

^{13}C NMR of compound **5b**

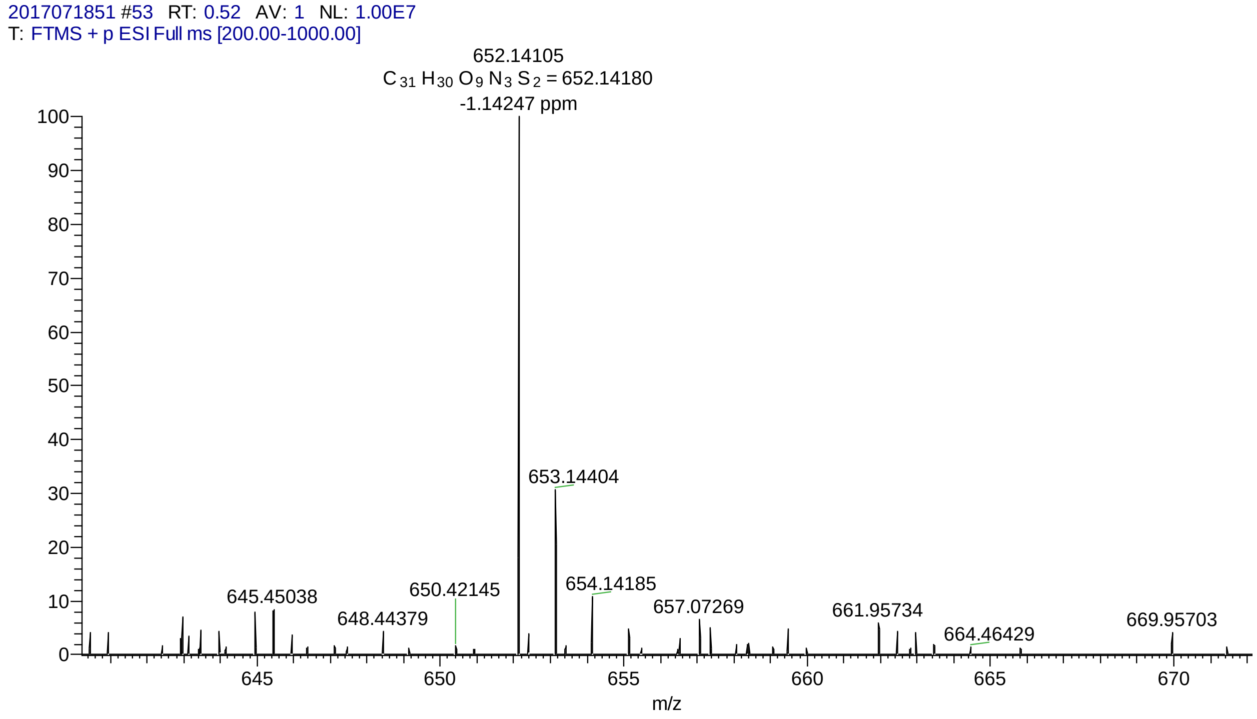

HRMS of compound **5b**

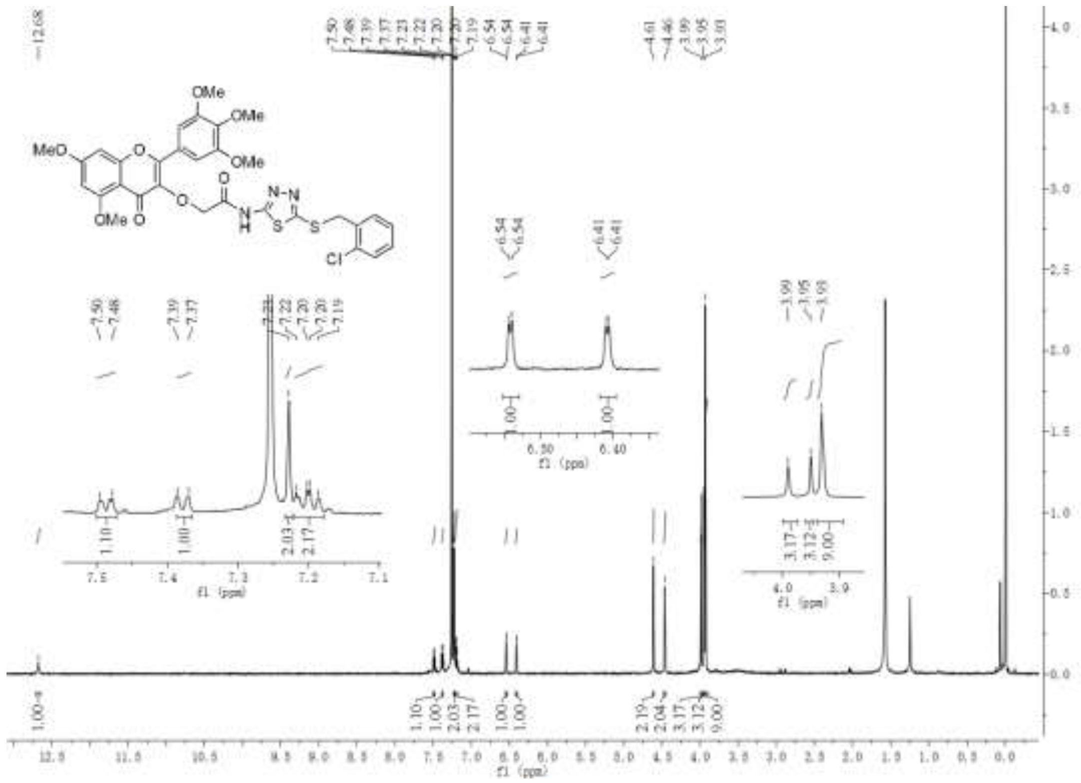

^{1}H NMR of compound **5c**

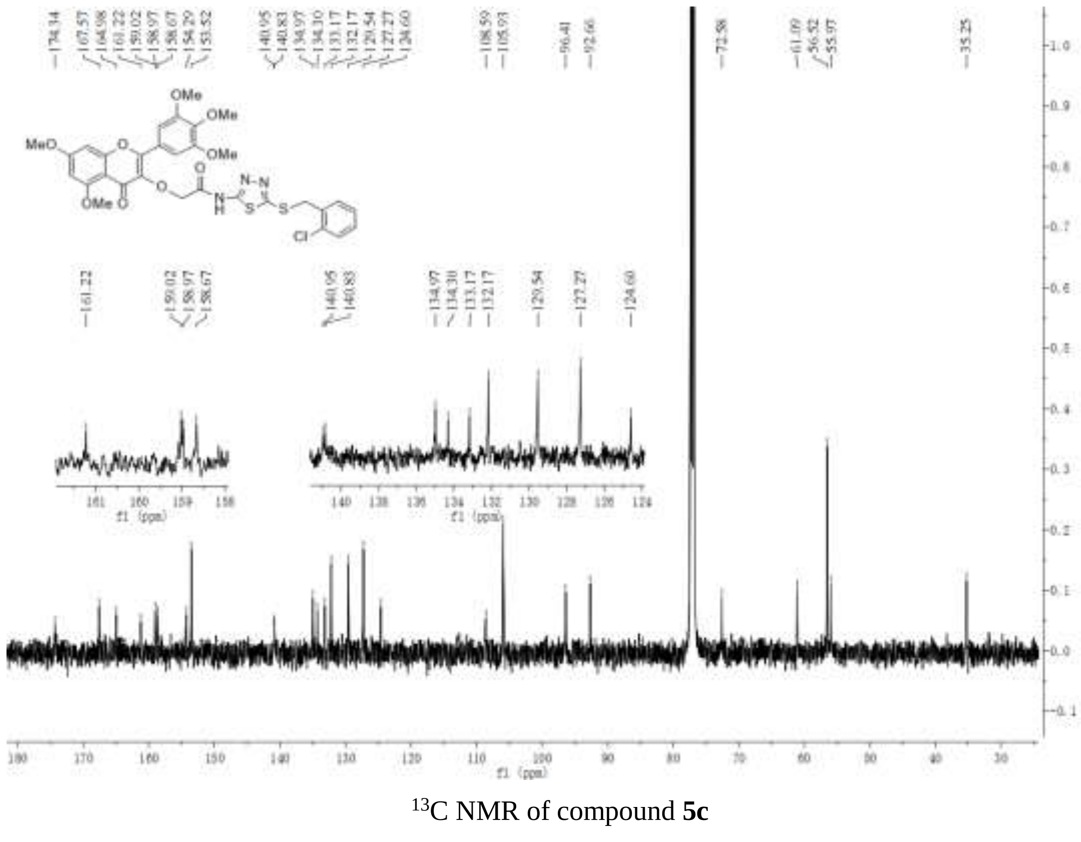

^{13}C NMR of compound **5c**

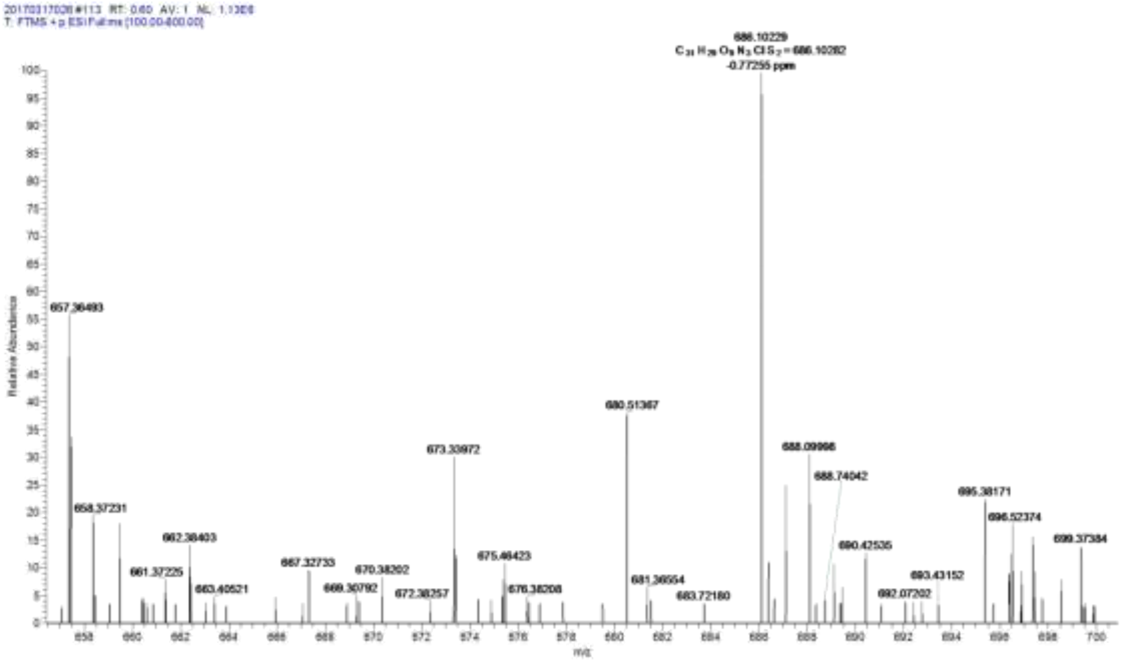

HRMS of compound **5c**

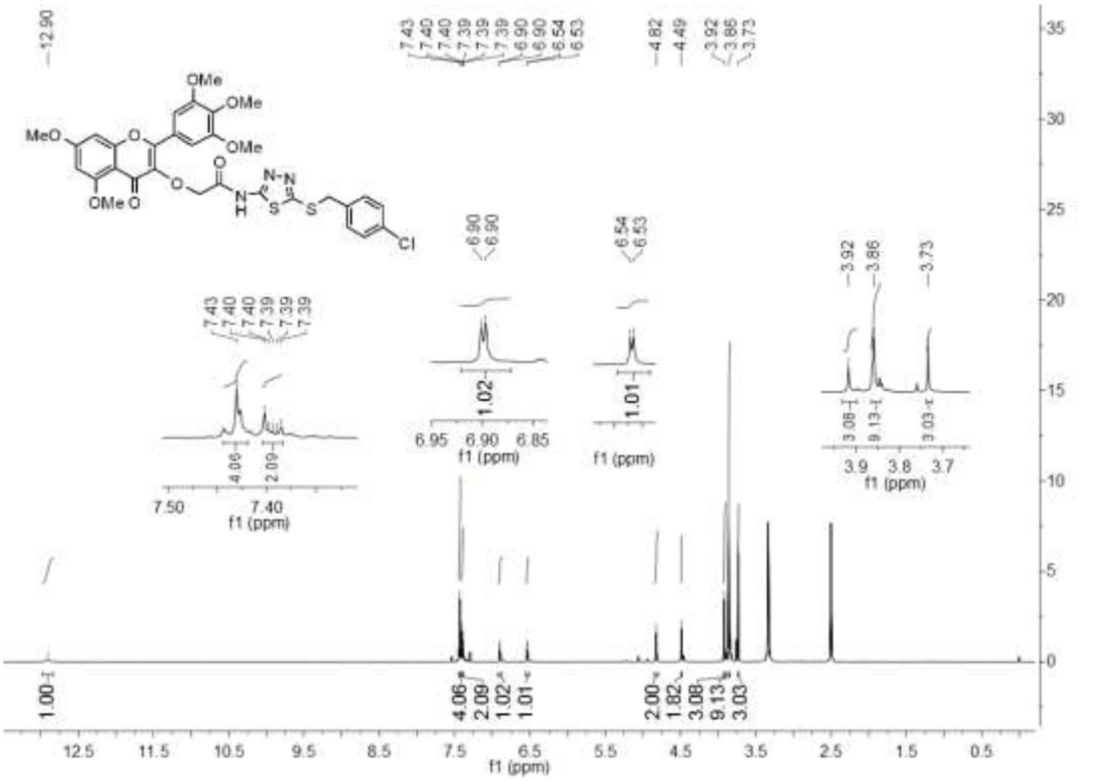

^{1}H NMR of compound **5d**

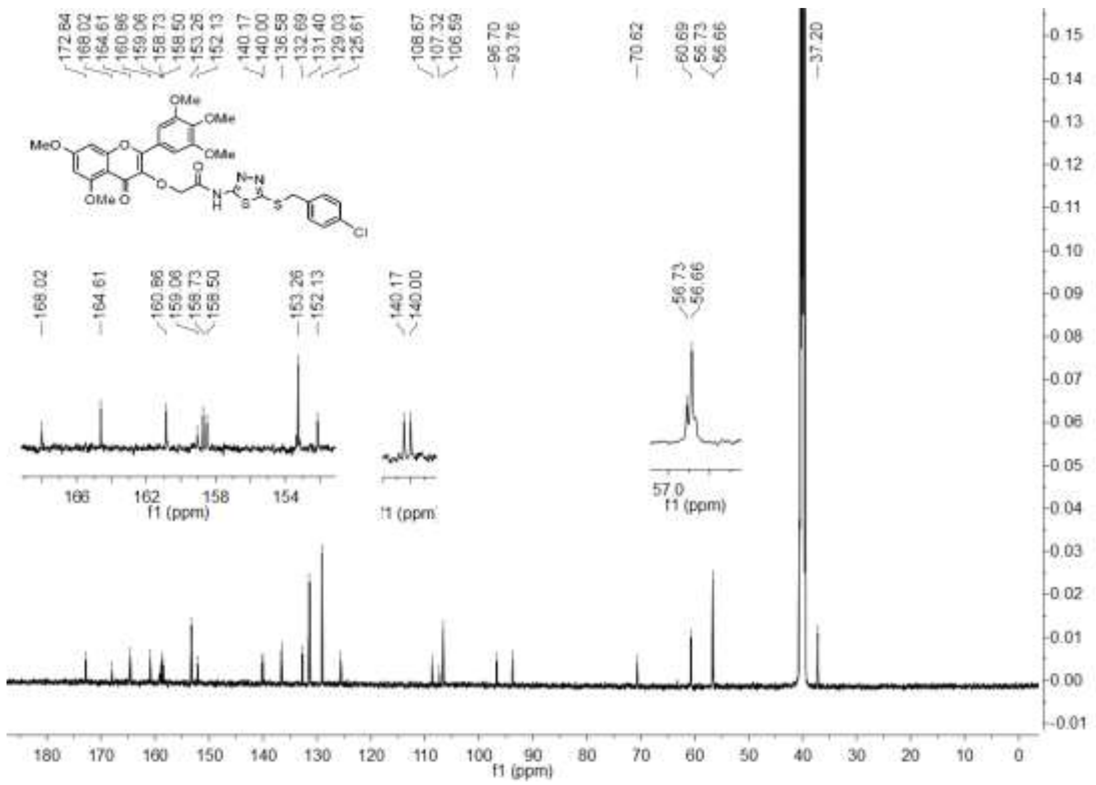

^{13}C NMR of compound **5d**

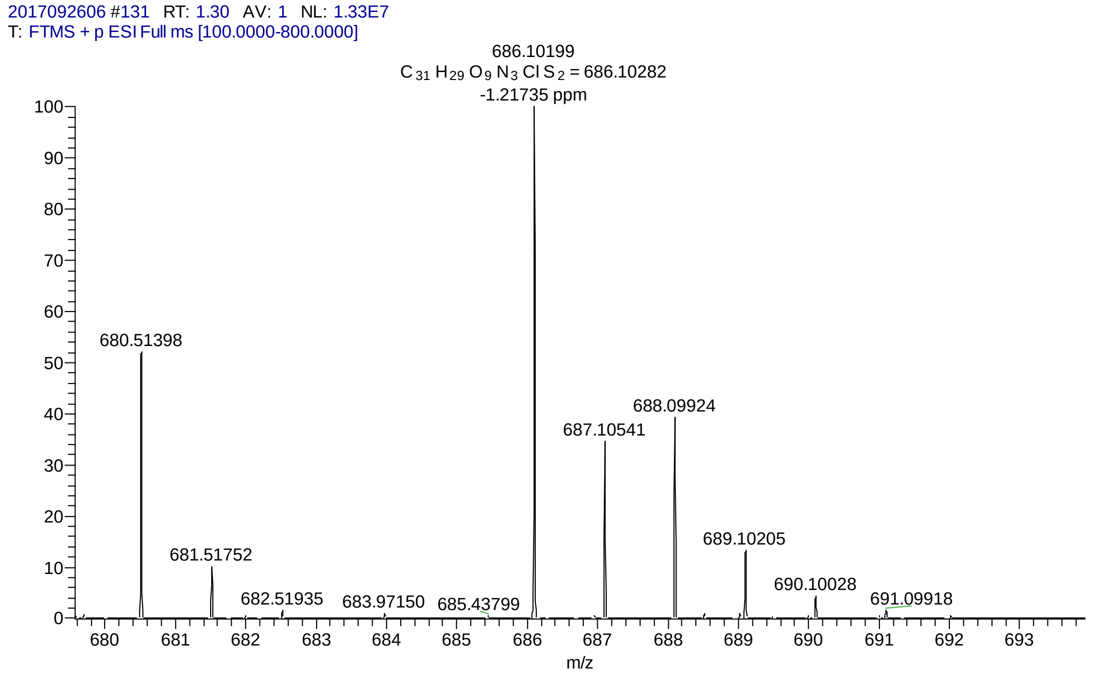

HRMS of compound **5d**

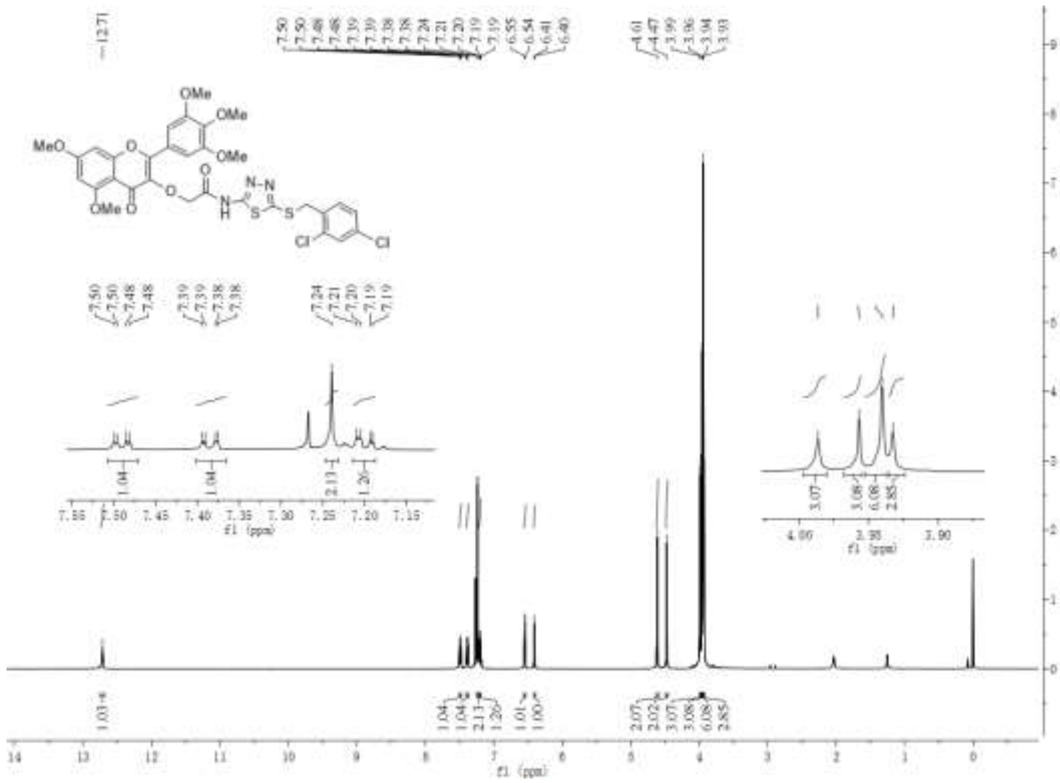

^{1}H NMR of compound **5e**

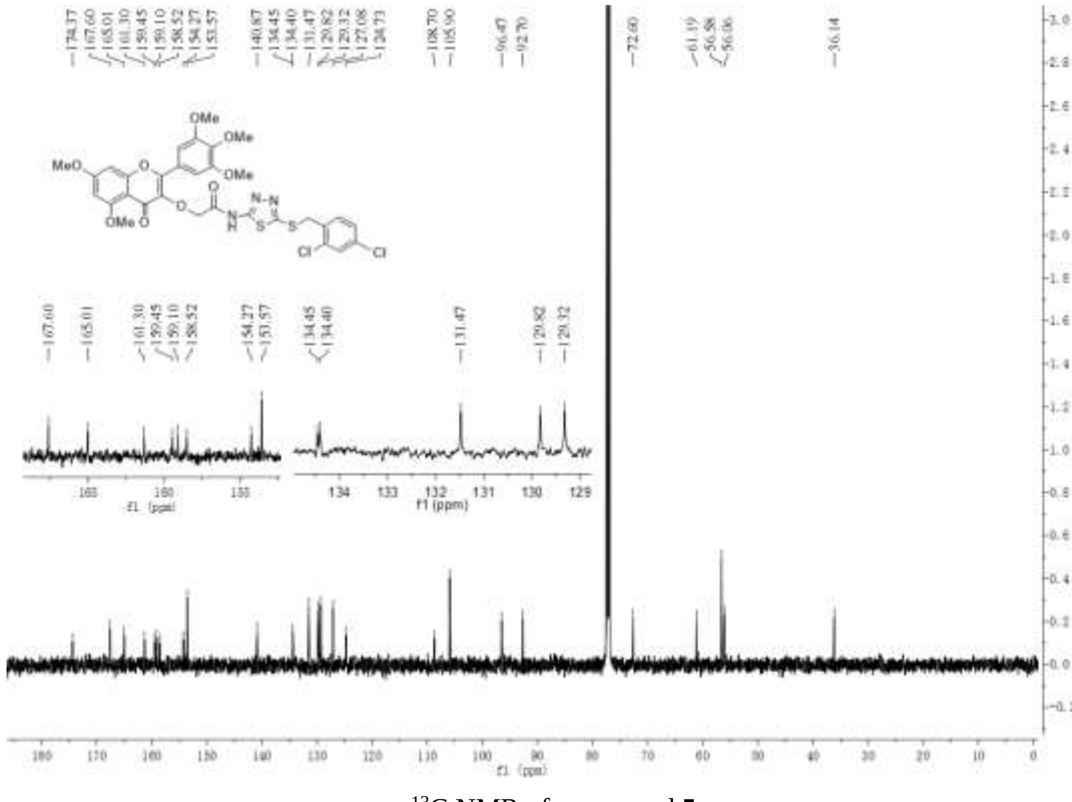

^{13}C NMR of compound **5e**

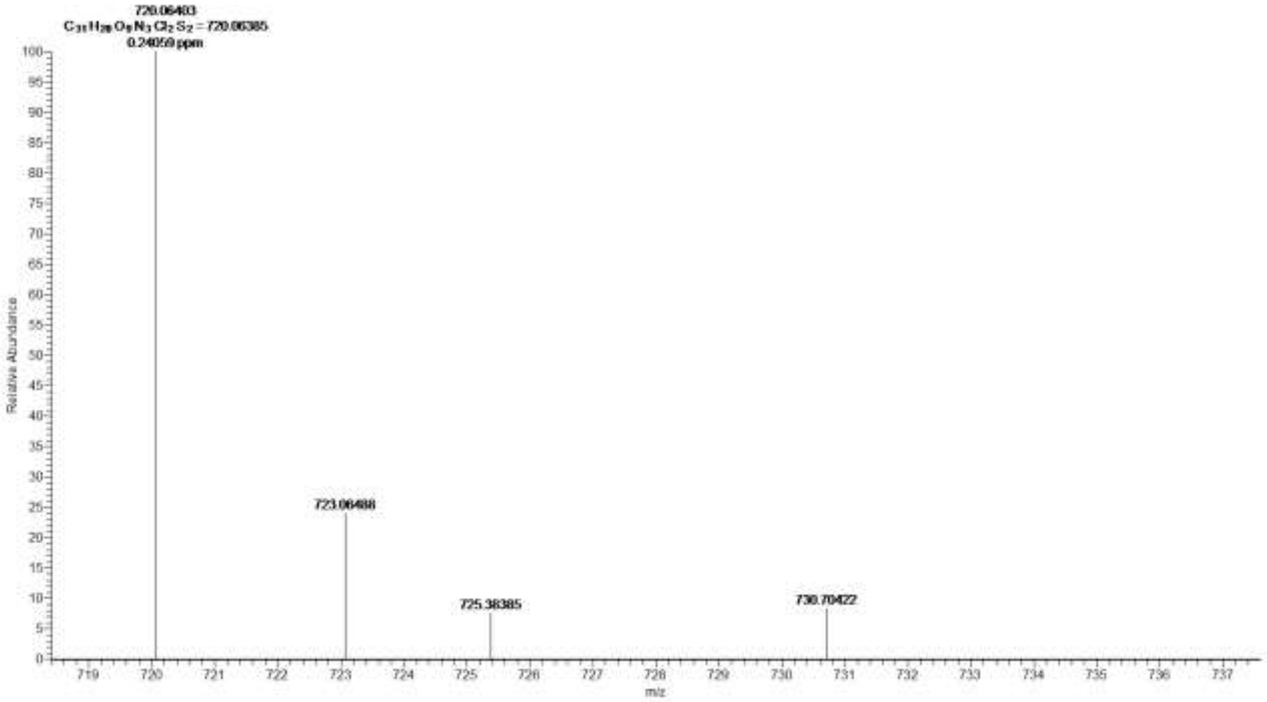

HRMS of compound **5e**

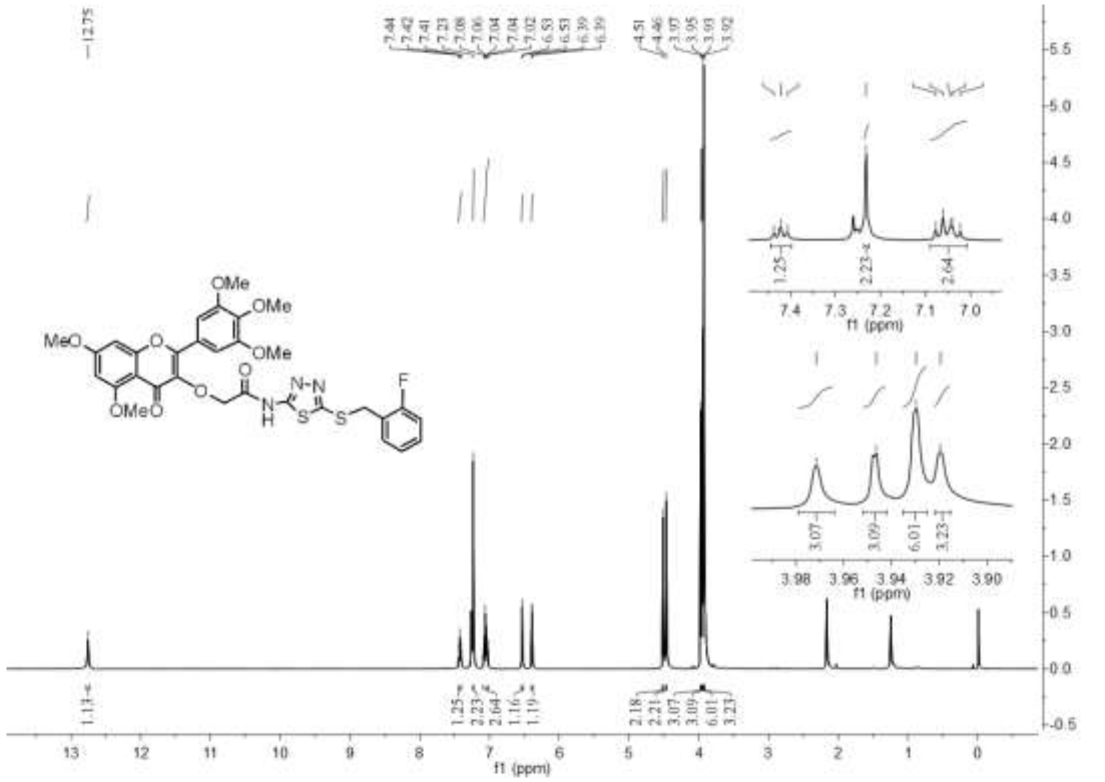

^{1}H NMR of compound **5f**

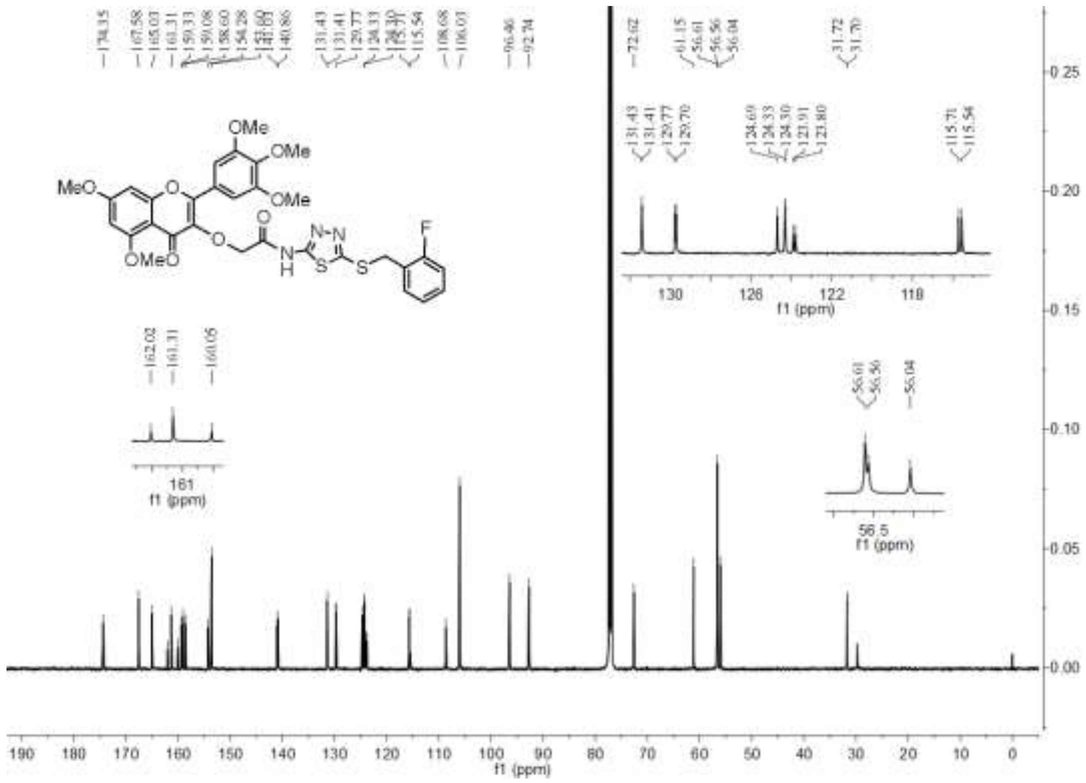

^{13}C NMR of compound **5f**

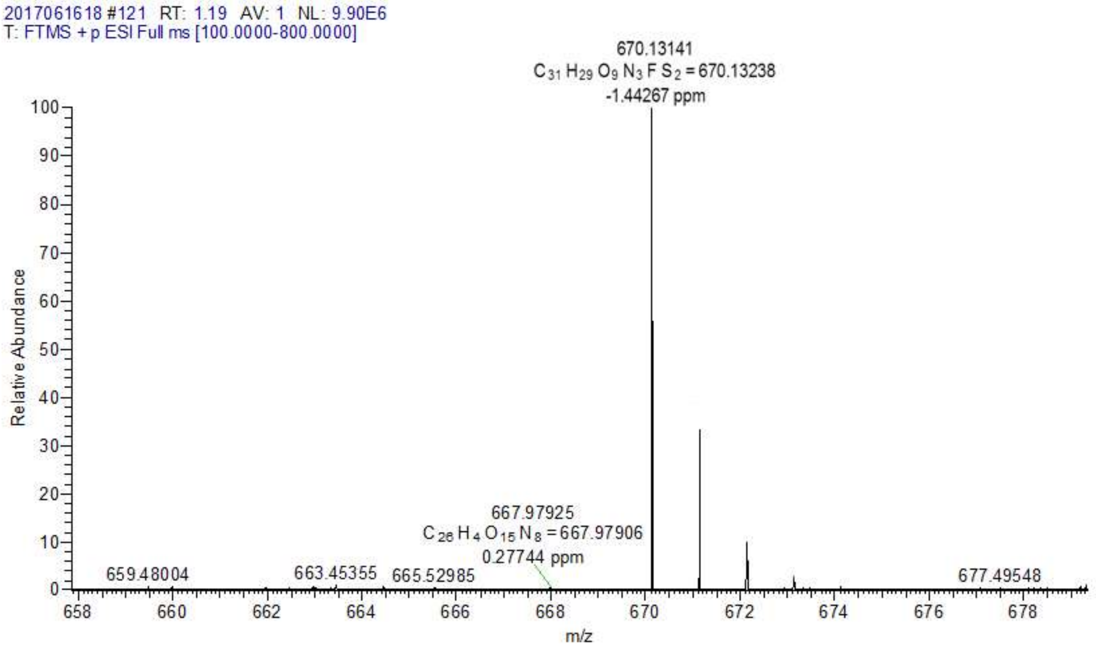

HRMS of compound **5f**

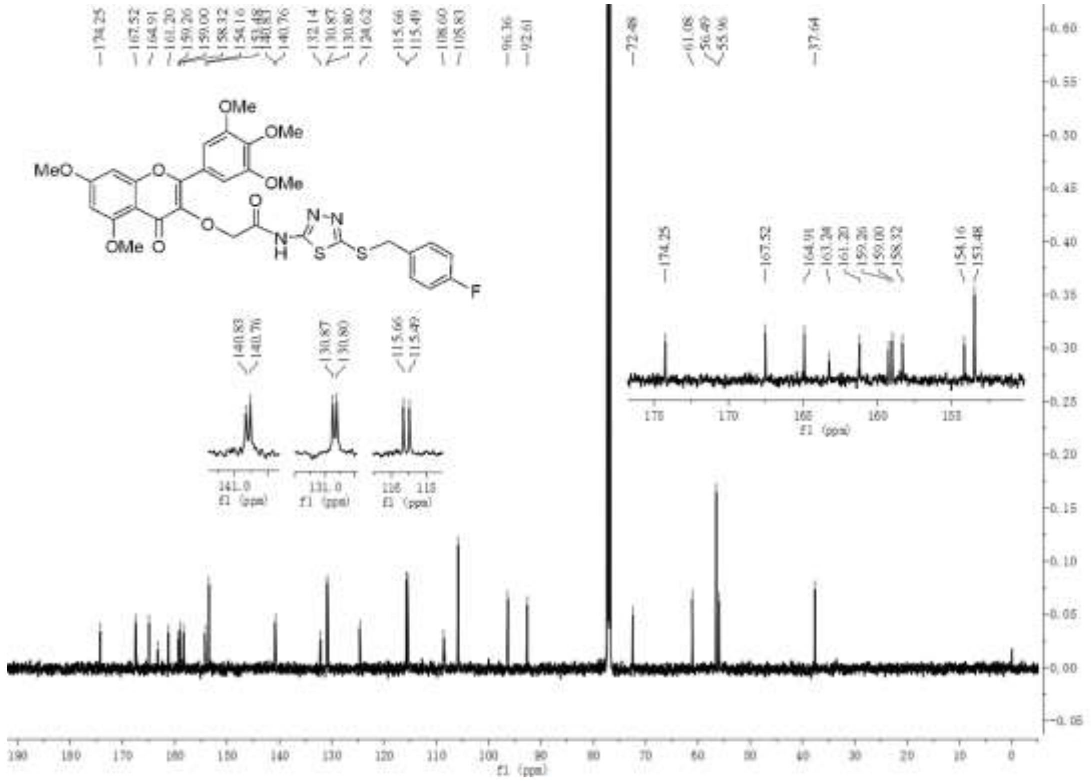

^{1}H NMR of compound **5g**

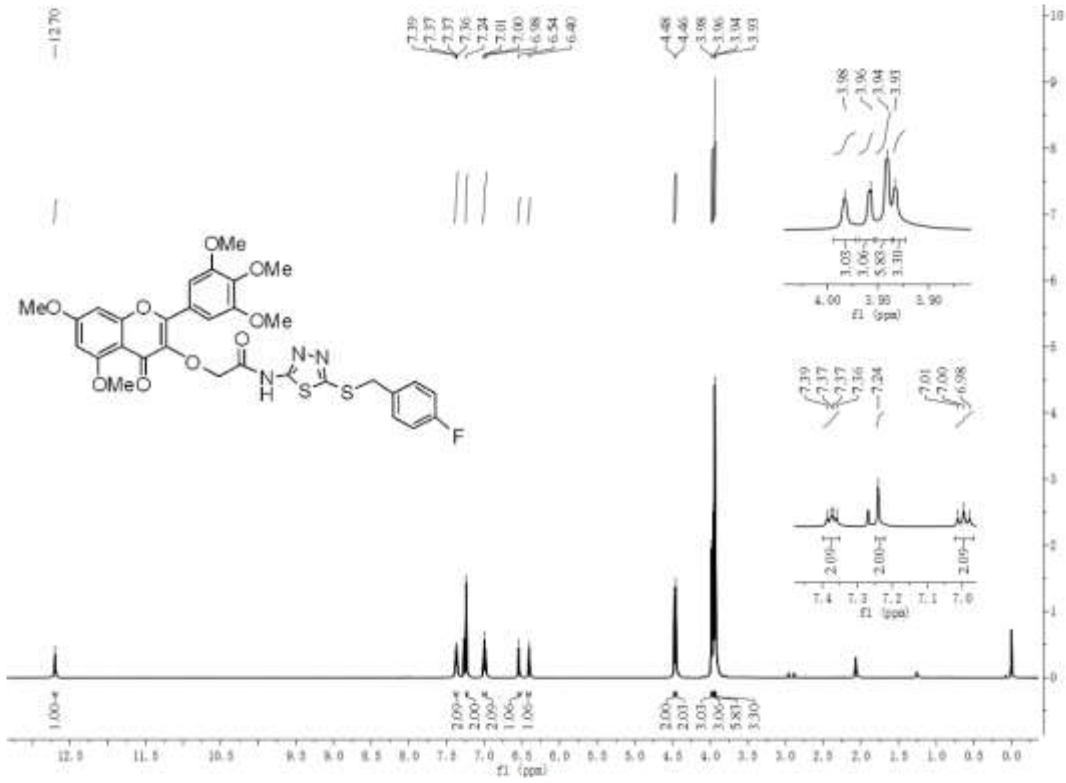

^{13}C NMR of compound **5g**

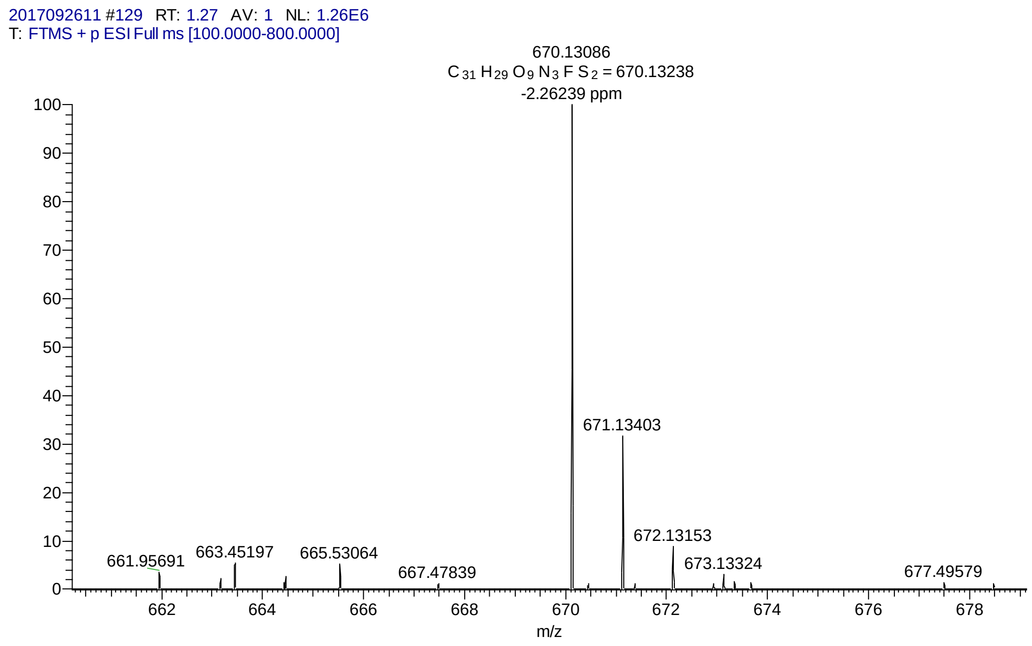

HRMS of compound **5a**

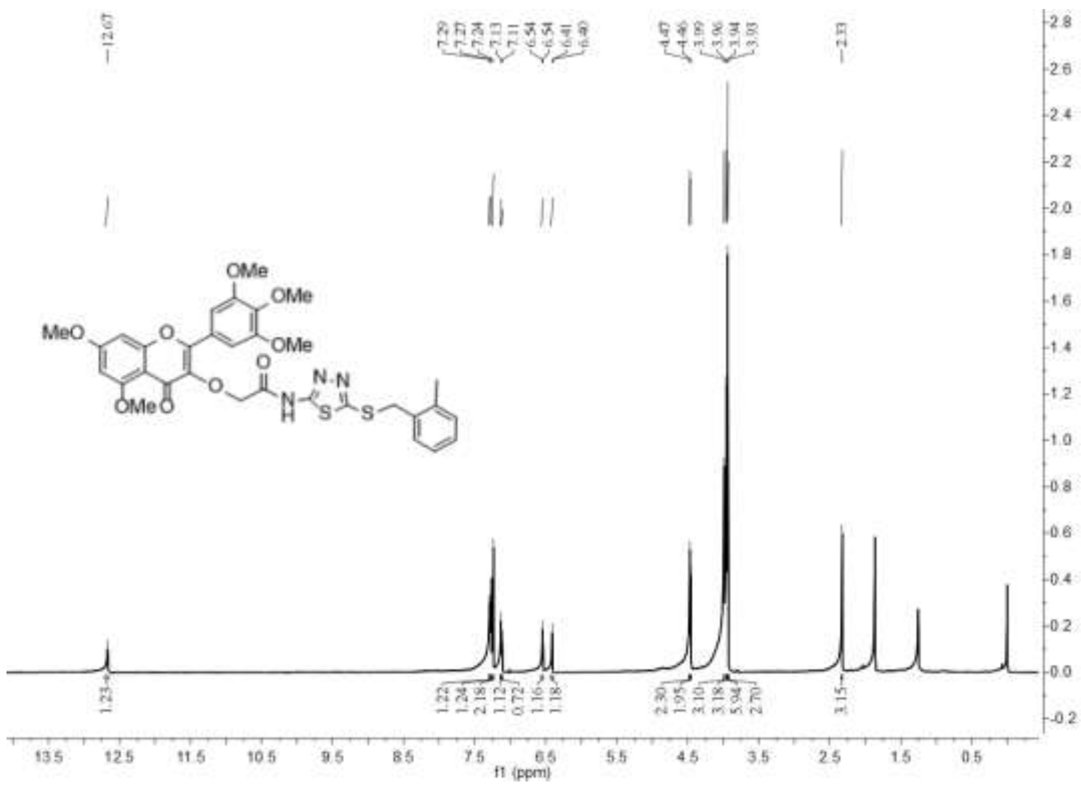

^{1}H NMR of compound **5h**

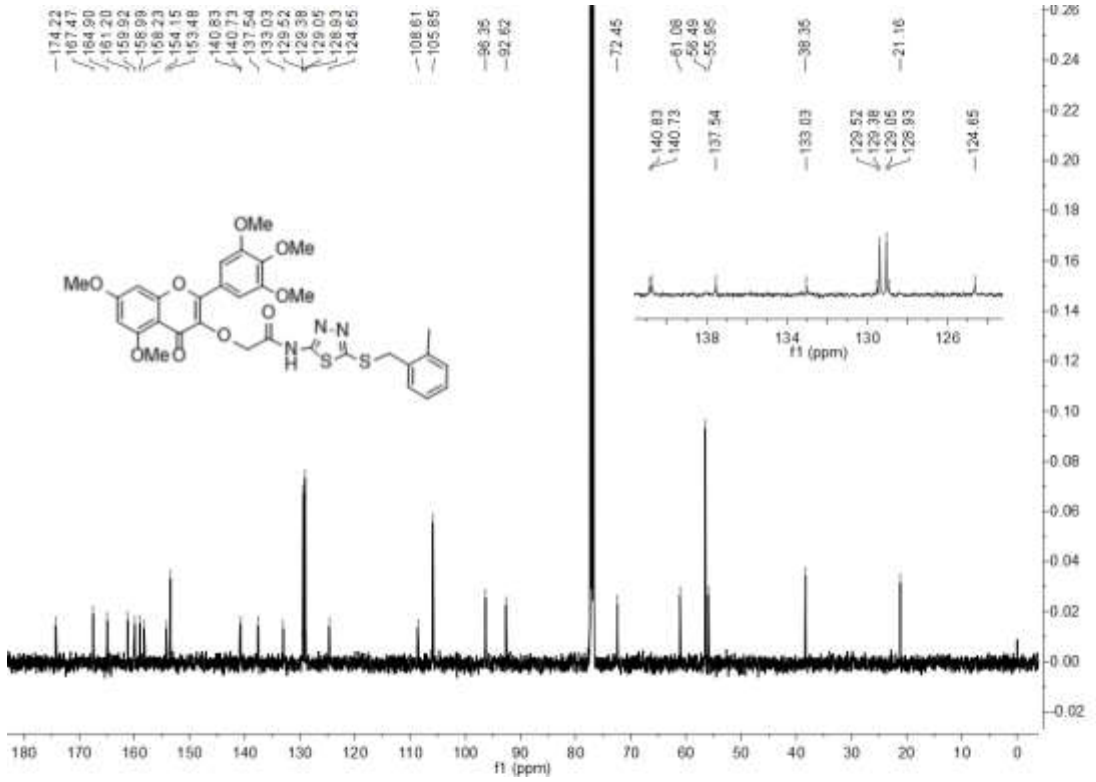

^{13}C NMR of compound **5h**

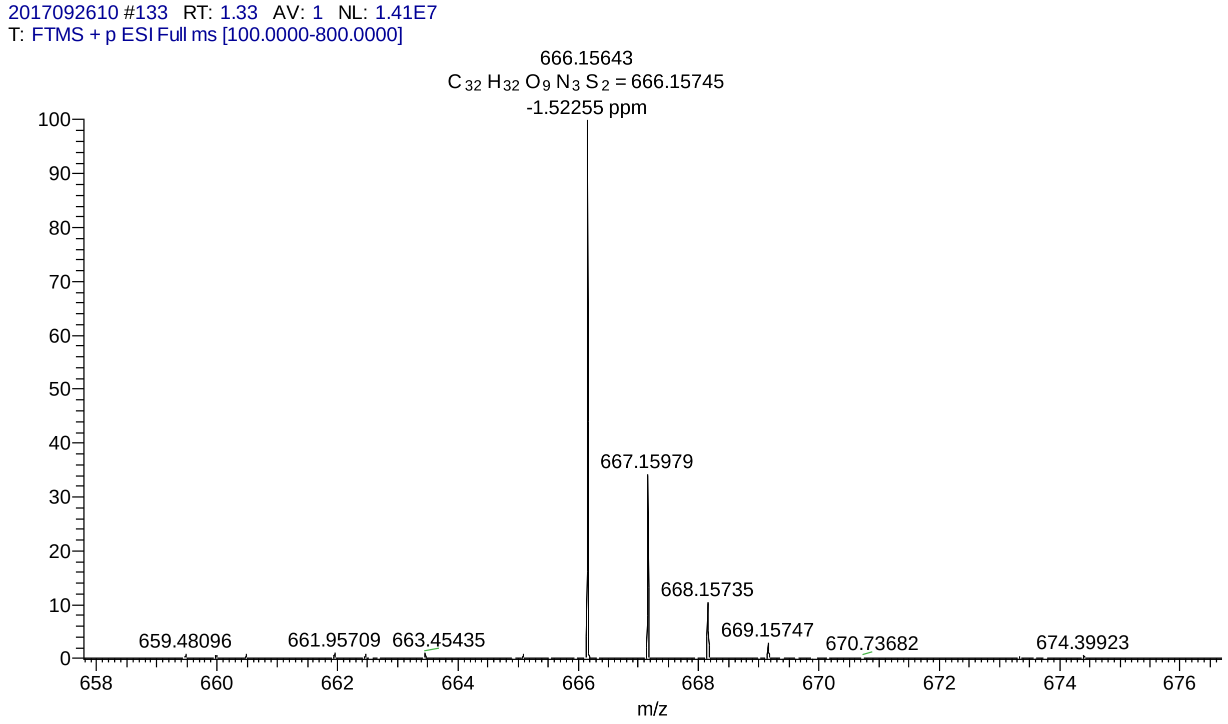

HRMS of compound **5h**

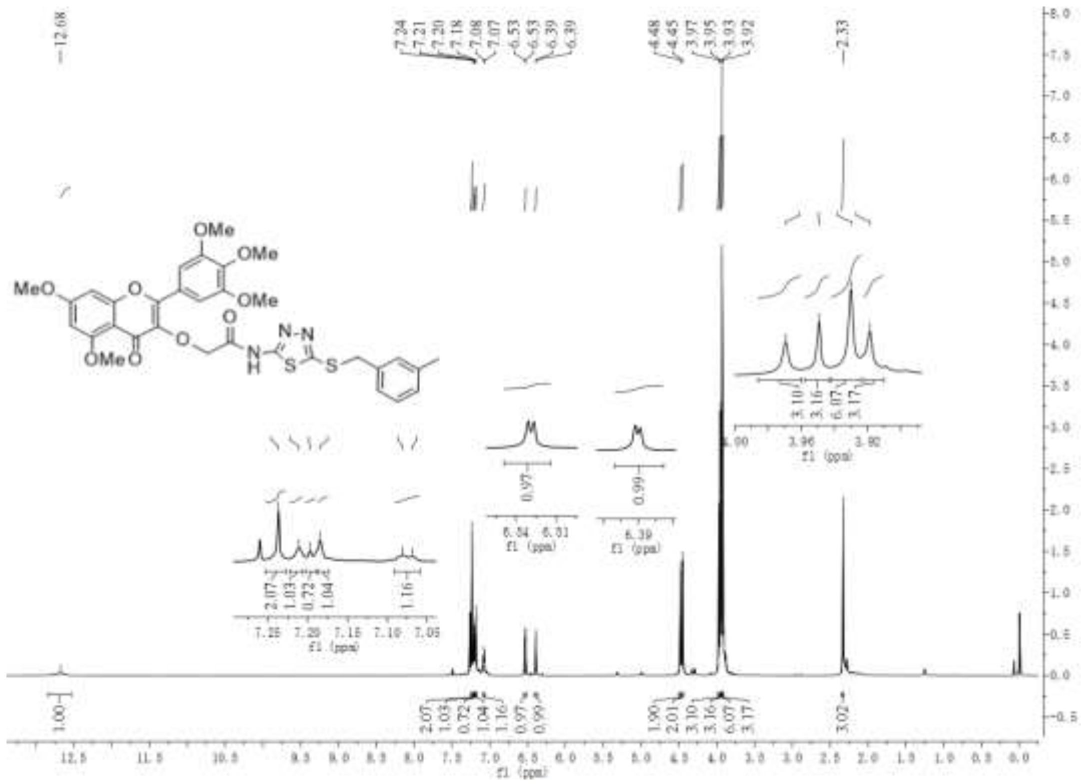

^{1}H NMR of compound **5i**

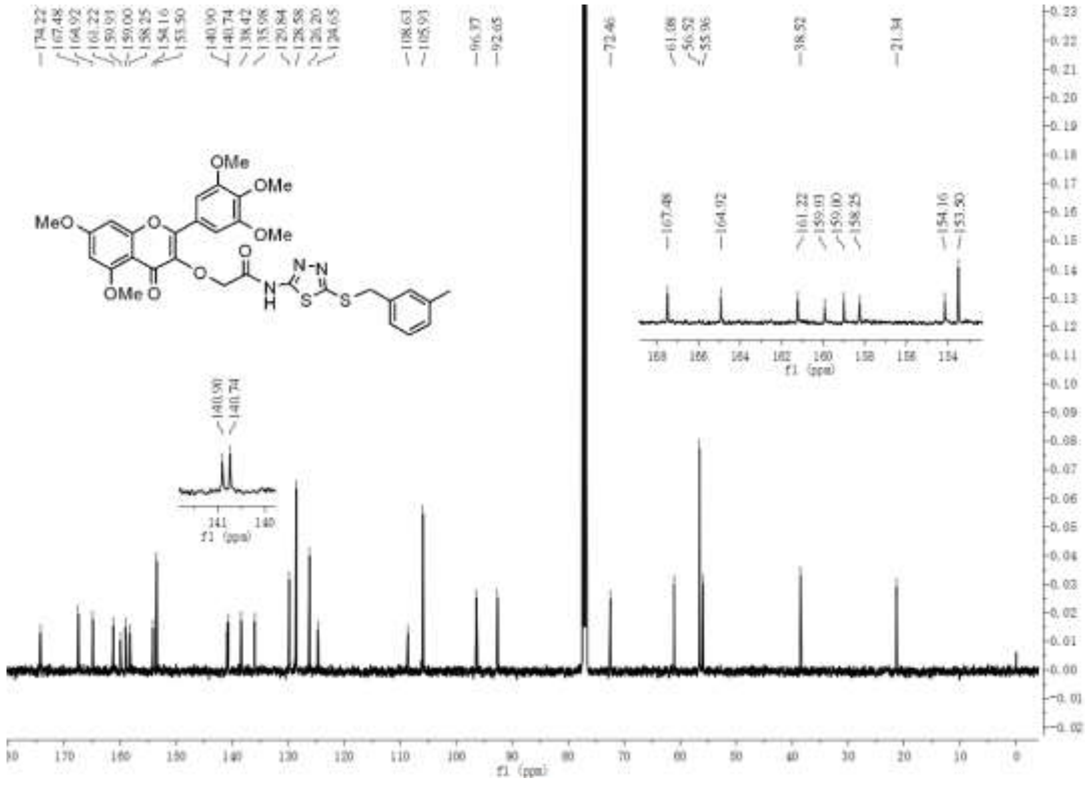

^{13}C NMR of compound **5i**

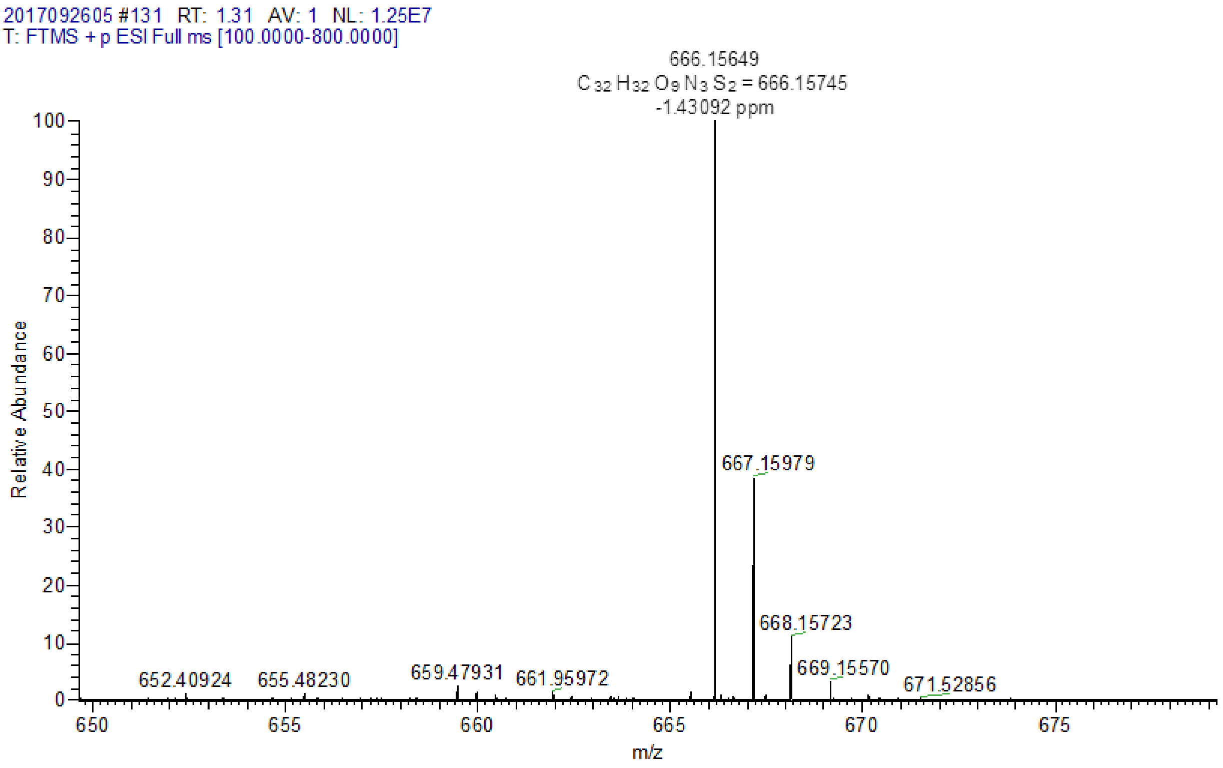

HRMS of compound **5i**

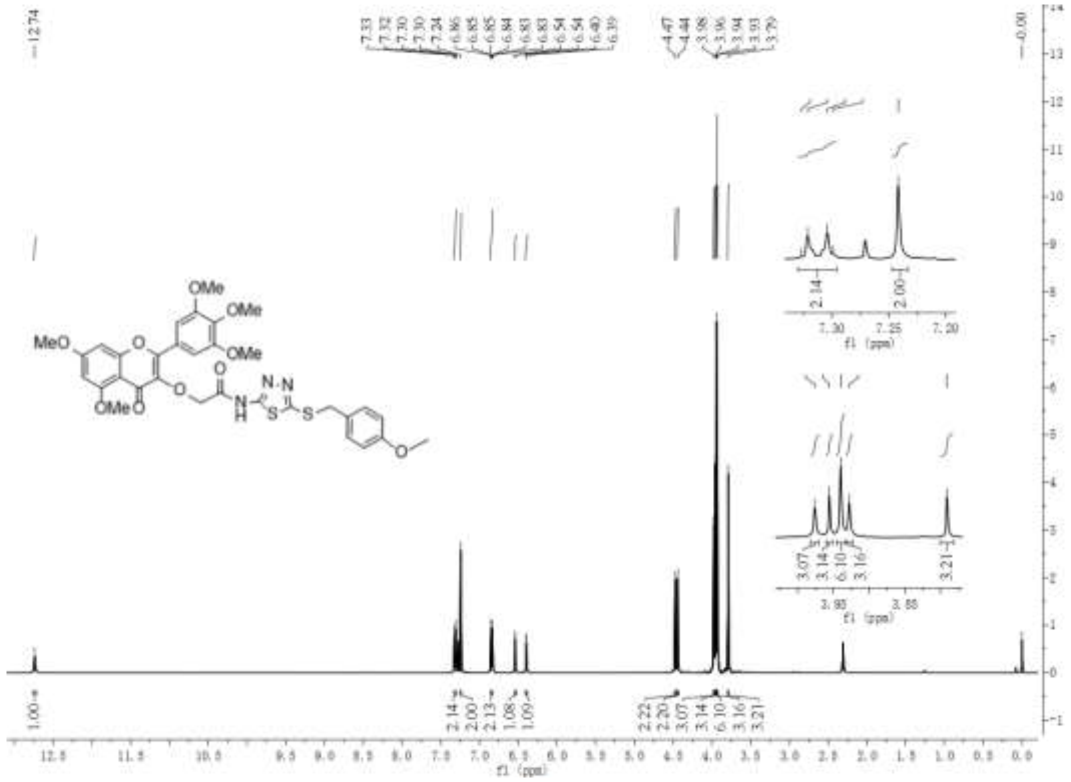

^{1}H NMR of compound **5j**

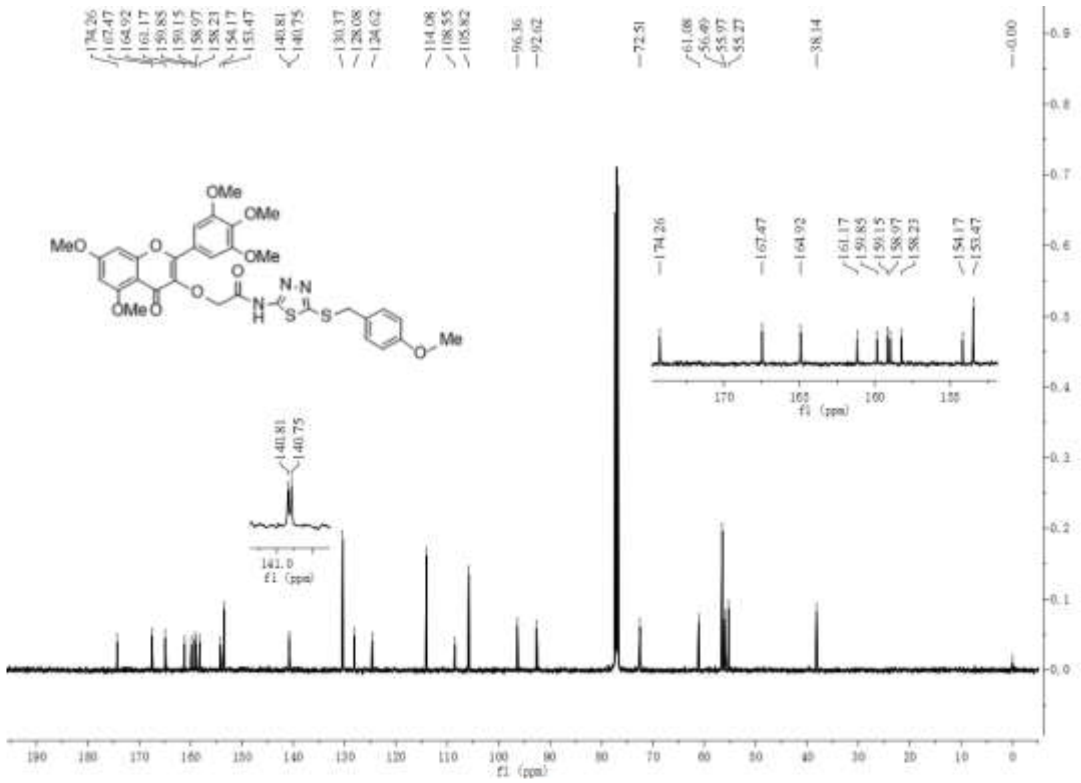

^{13}C NMR of compound **5j**

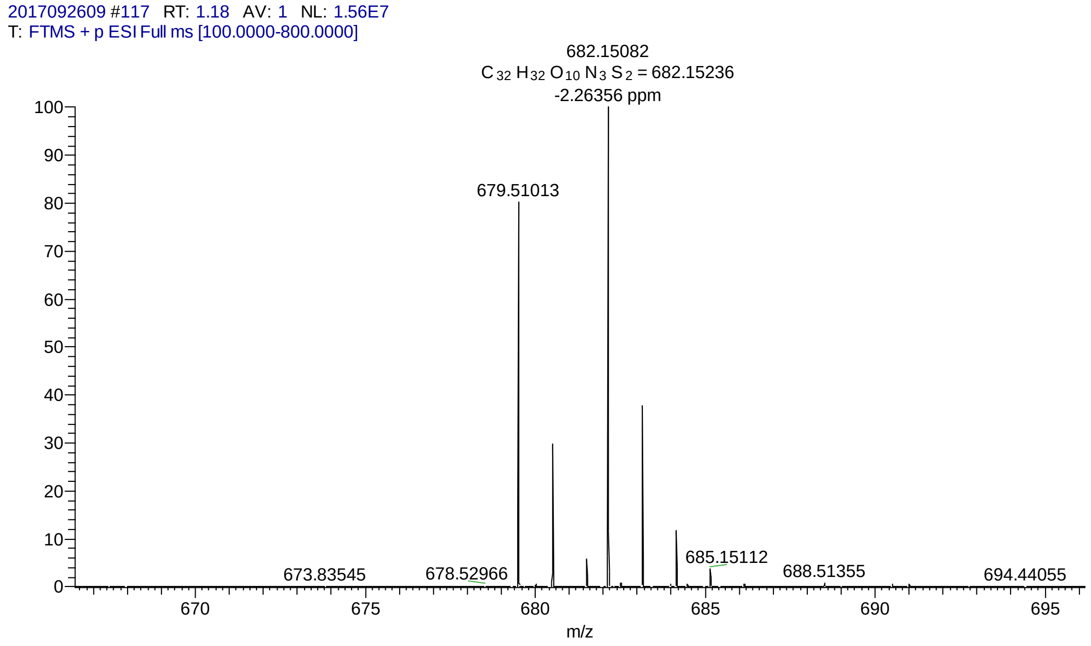

HRMS of compound **5j**

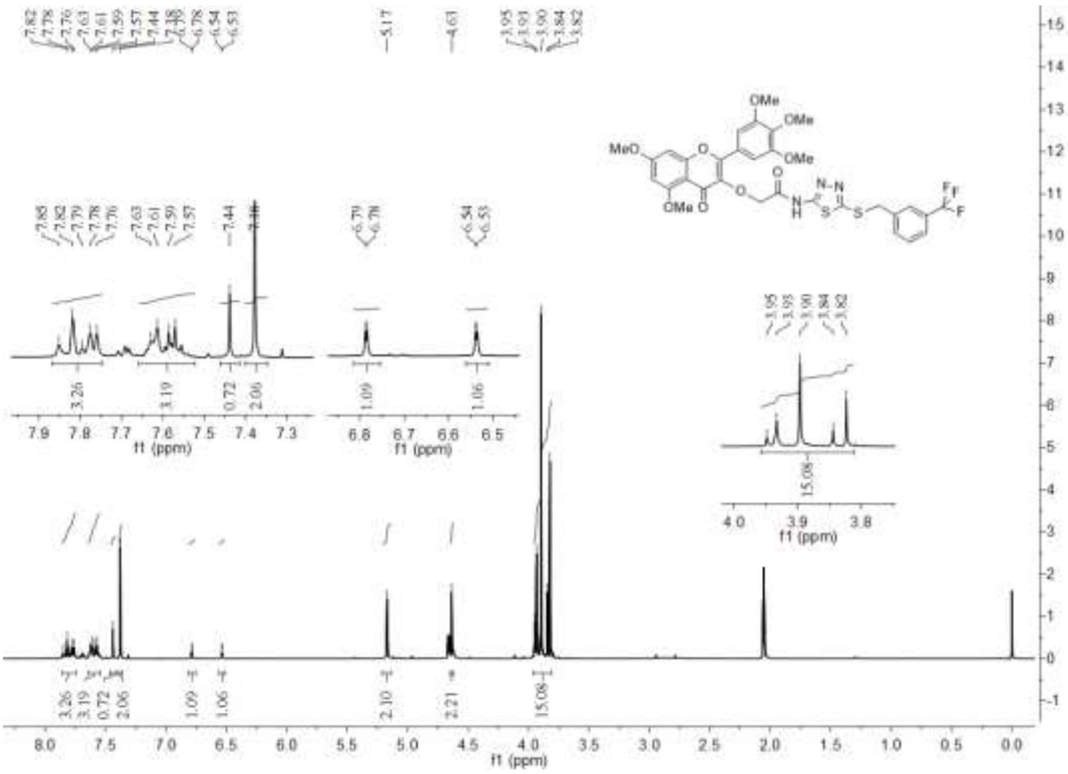

^{1}H NMR of compound **5k**

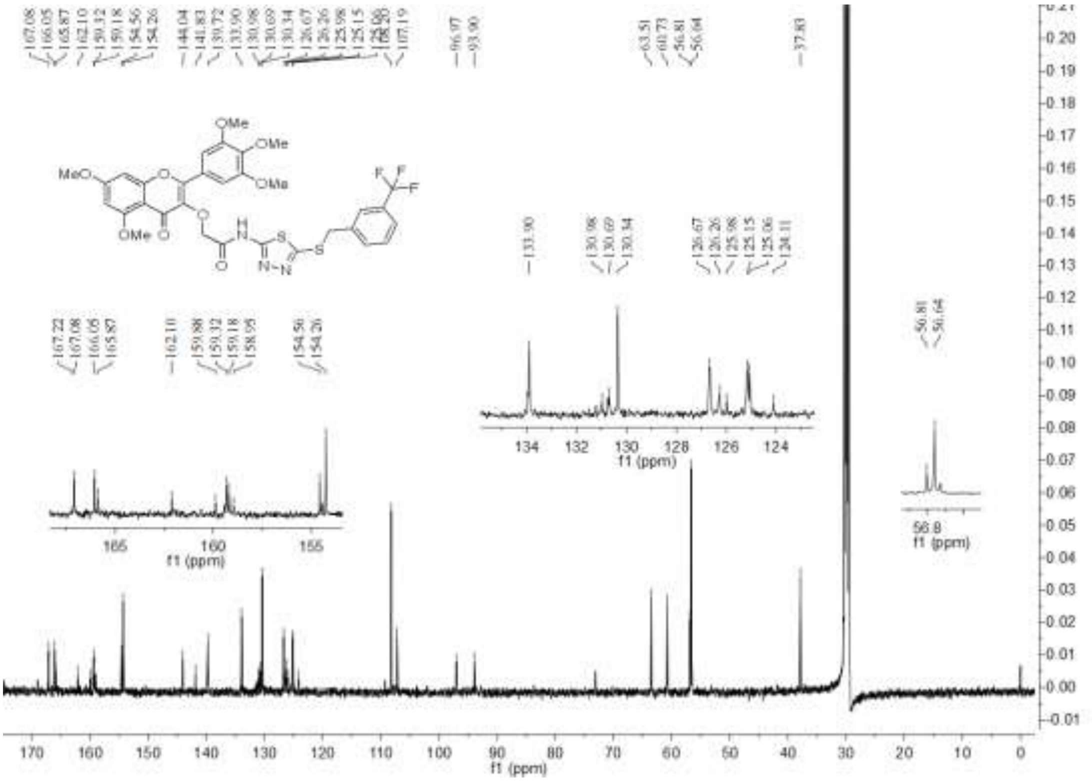

^{13}C NMR of compound **5k**

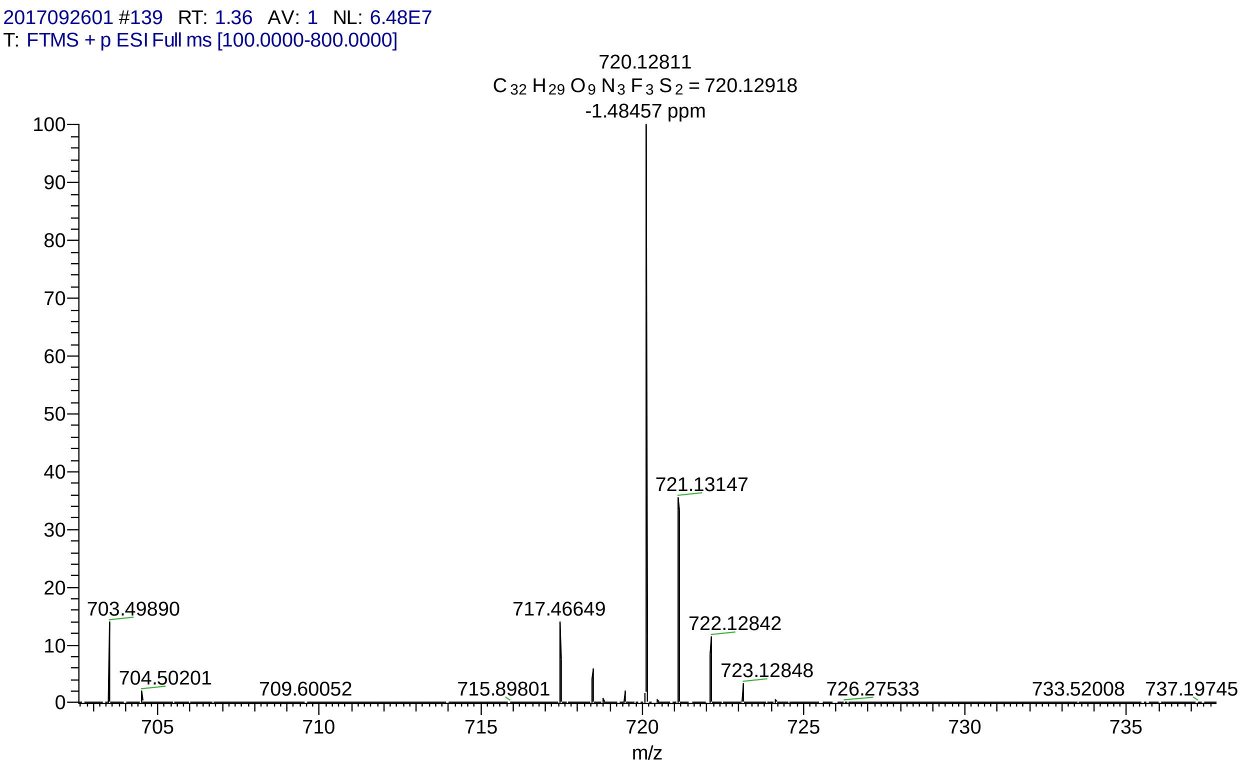

HRMS of compound **5k**

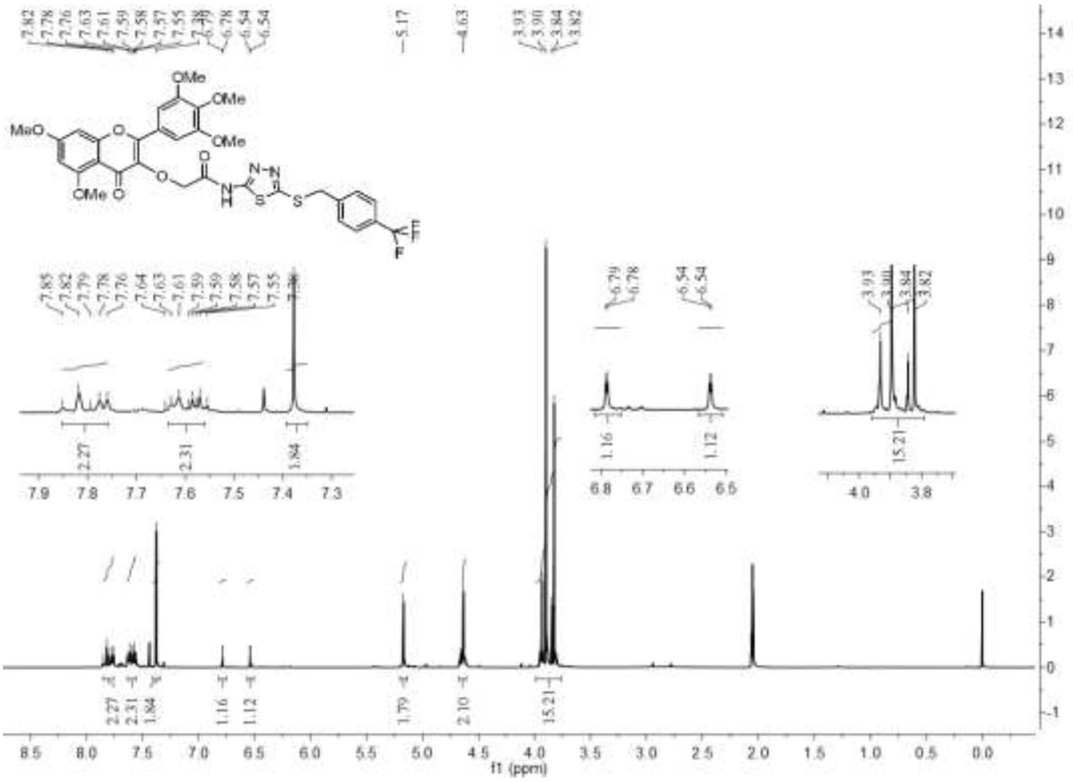

^{1}H NMR of compound **5l**

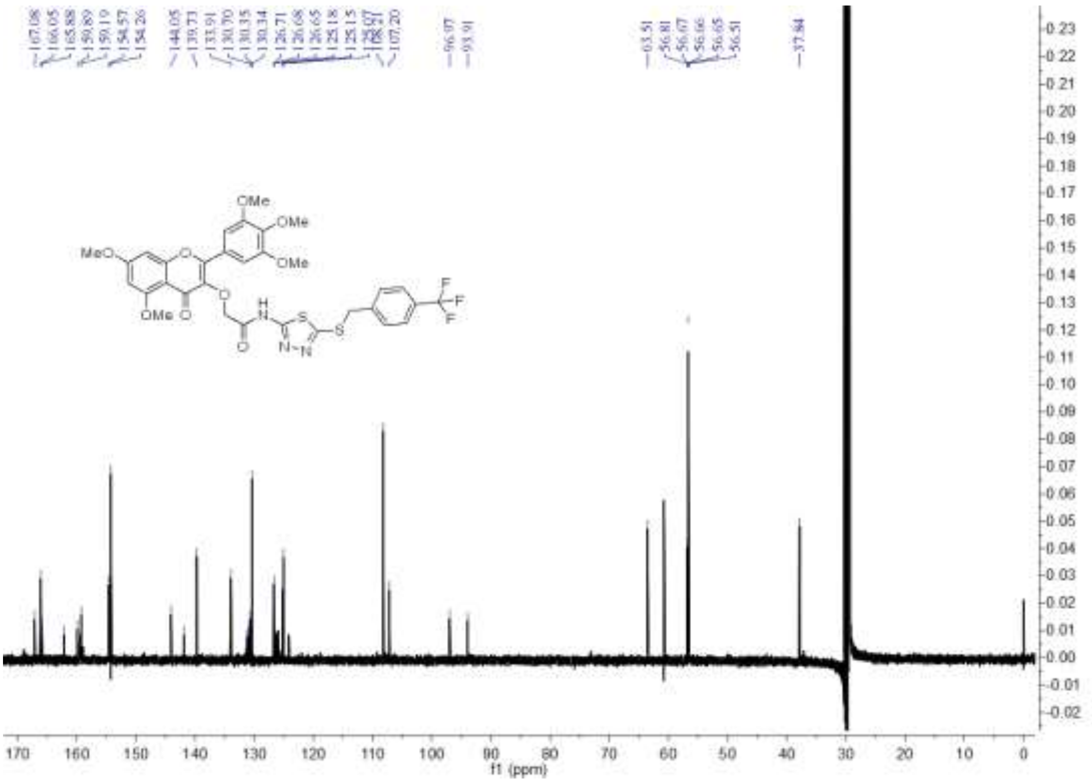

^{13}C NMR of compound **5l**

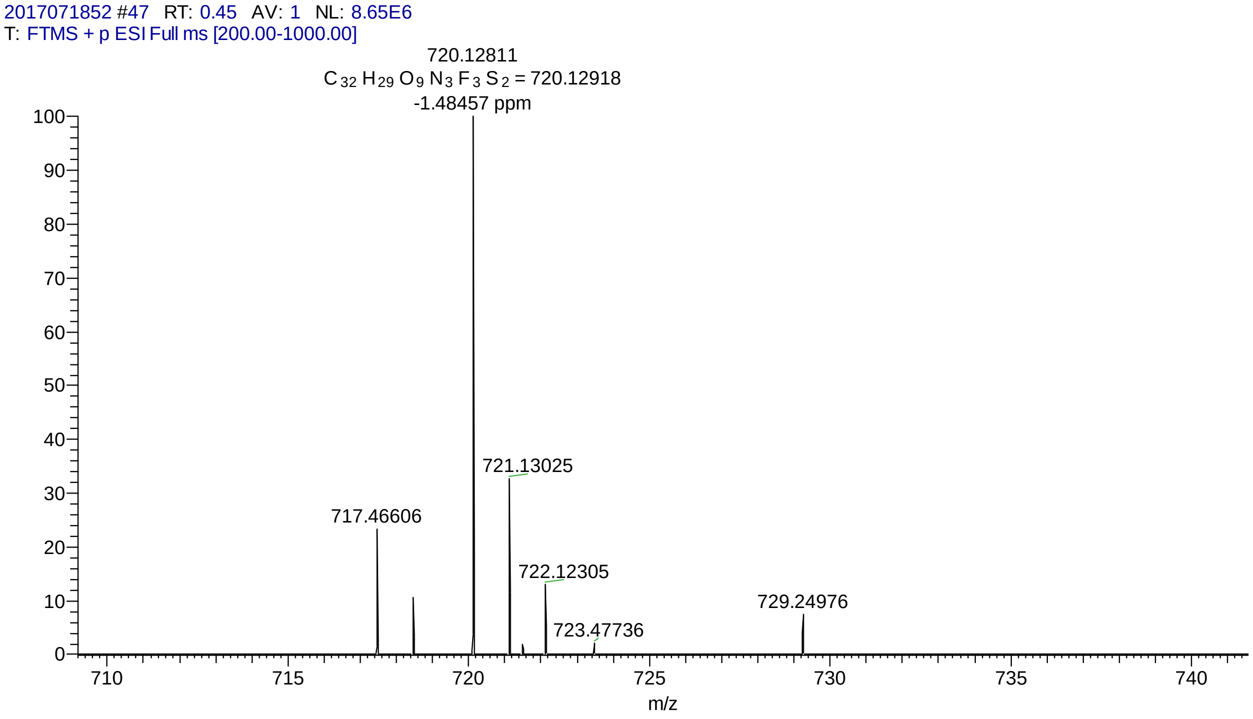

HRMS of compound **5l**

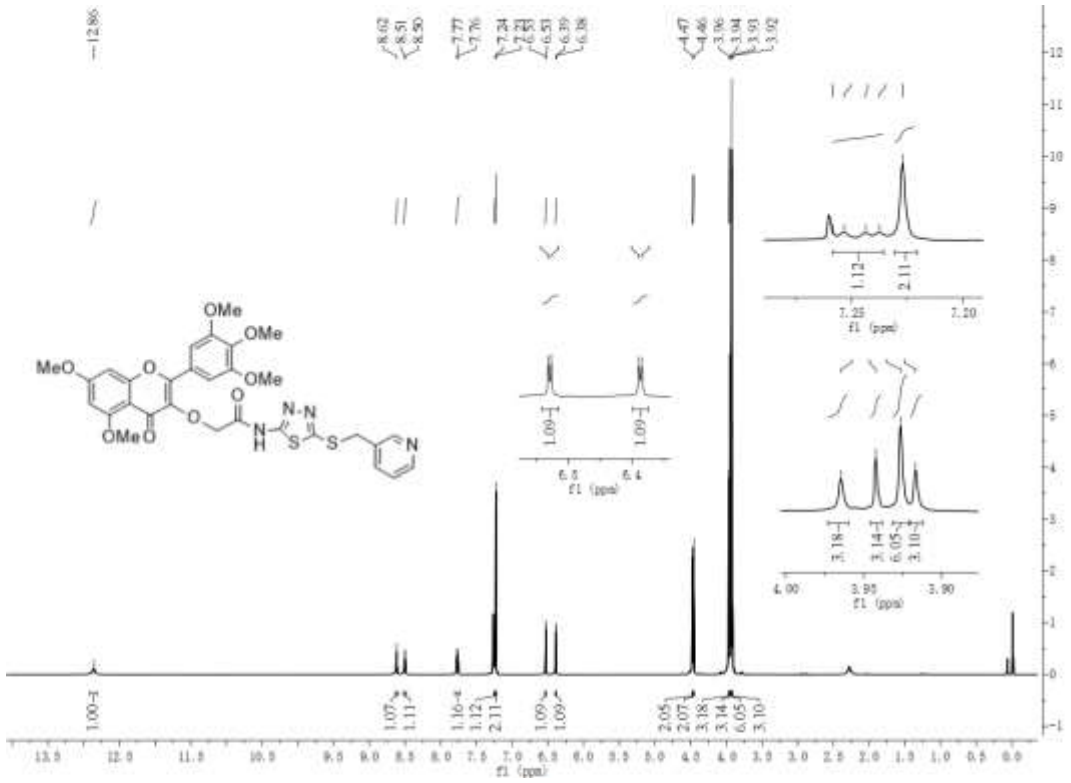

^{1}H NMR of compound **5m**

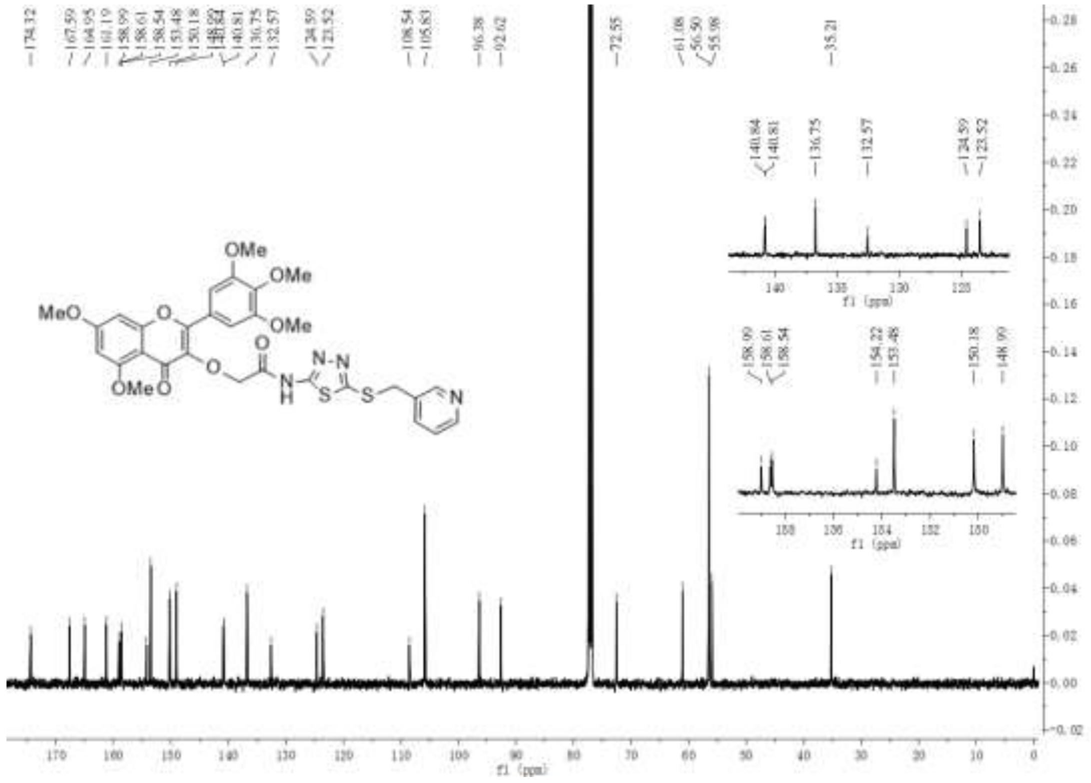

^{13}C NMR of compound **5m**

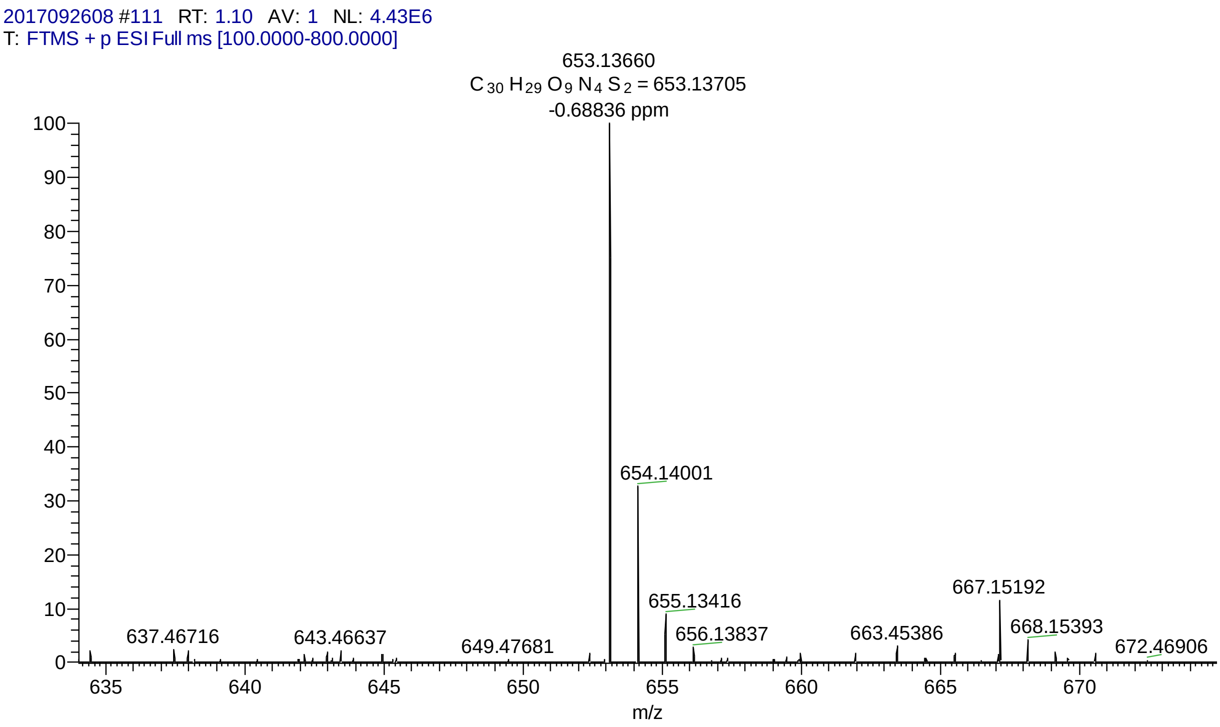

HRMS of compound **5m**

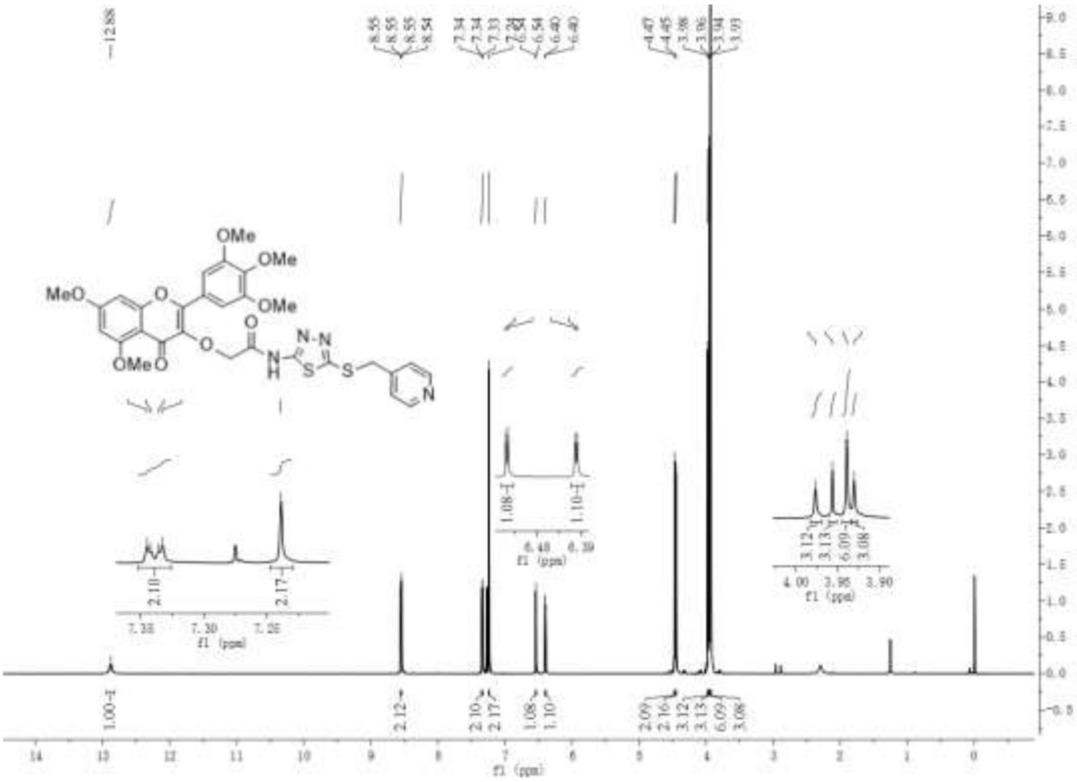

^{1}H NMR of compound **5n**

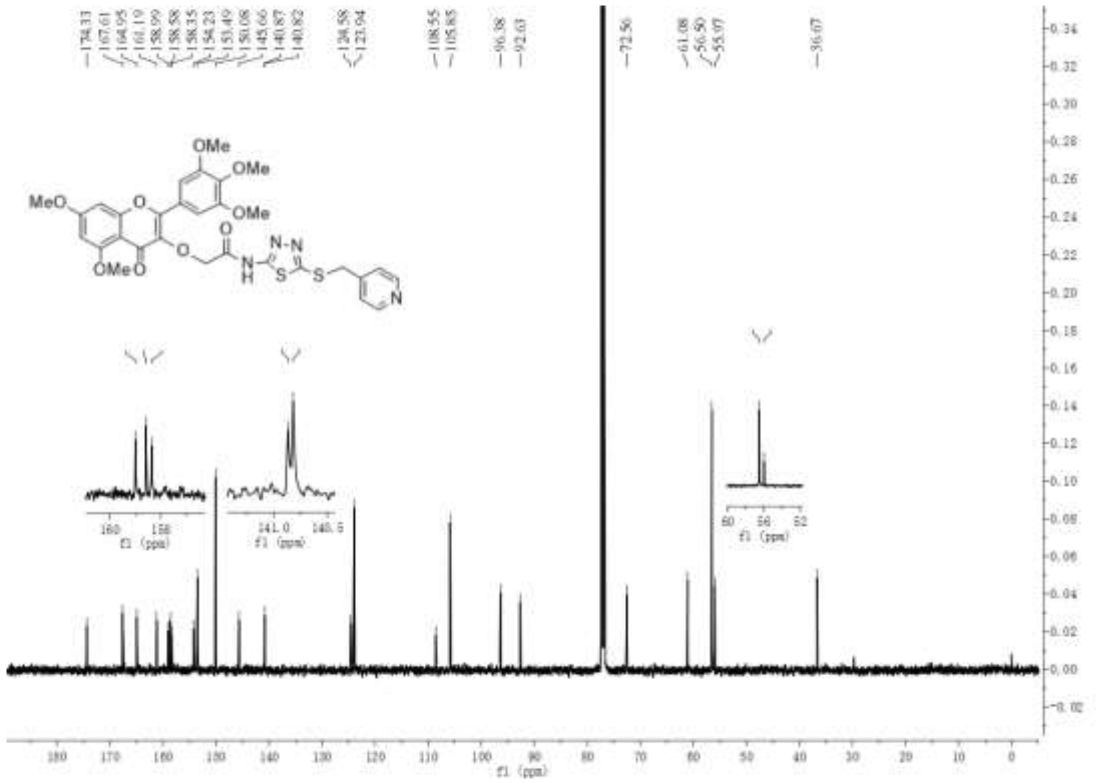

^{13}C NMR of compound **5n**

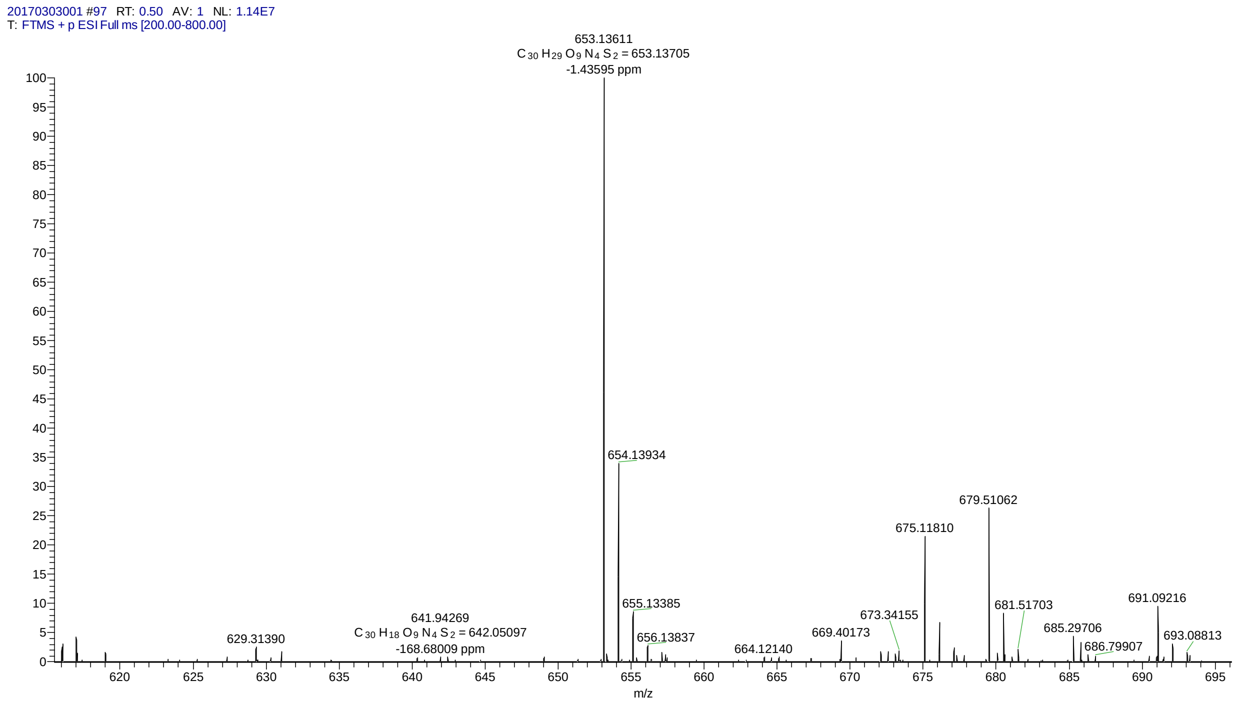

HRMS of compound **5n**

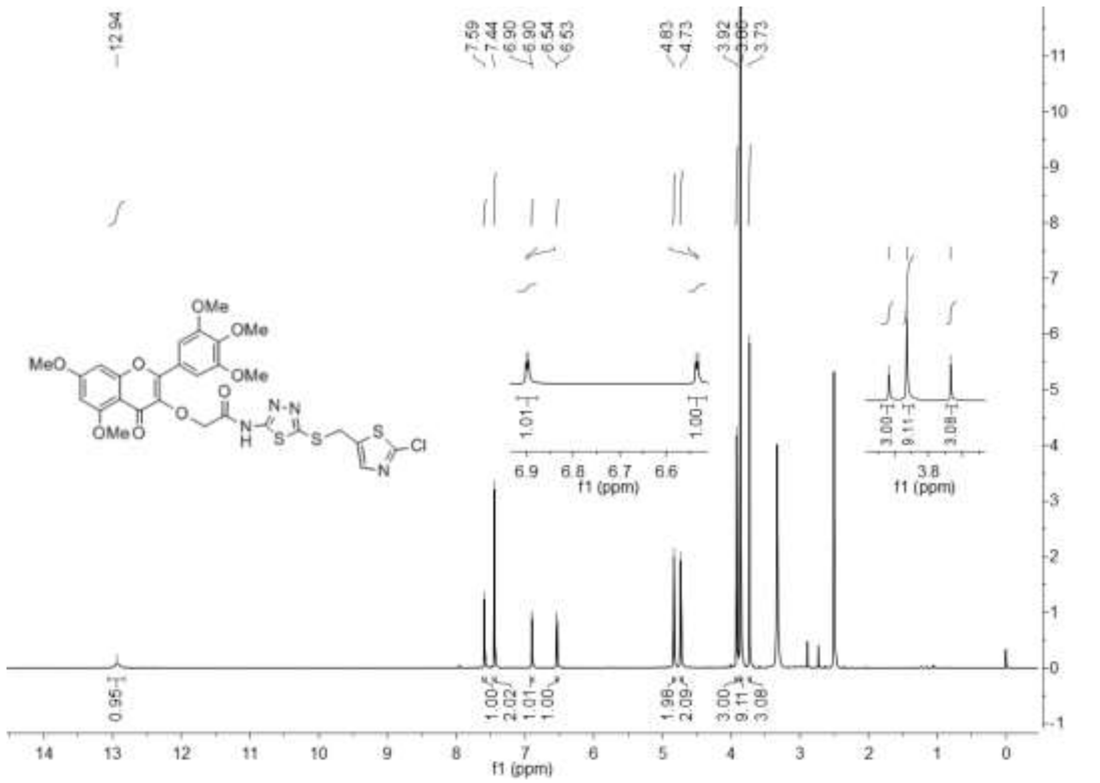

^{1}H NMR of compound **5o**

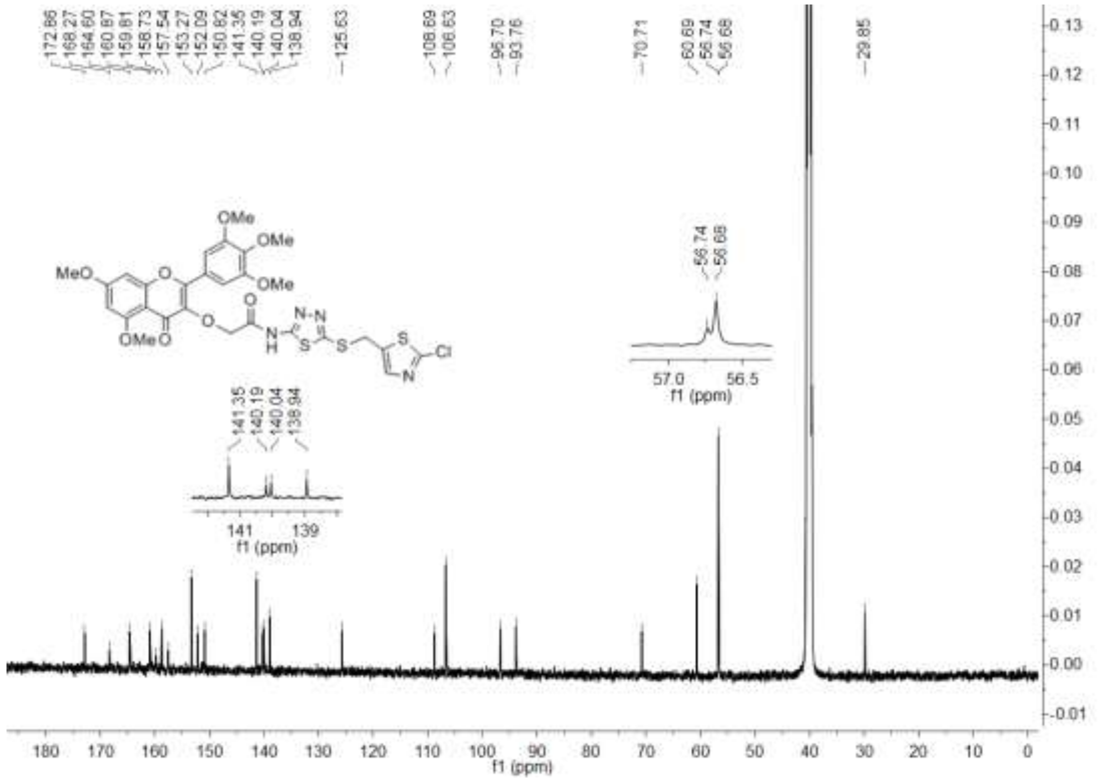

^{13}C NMR of compound **5o**

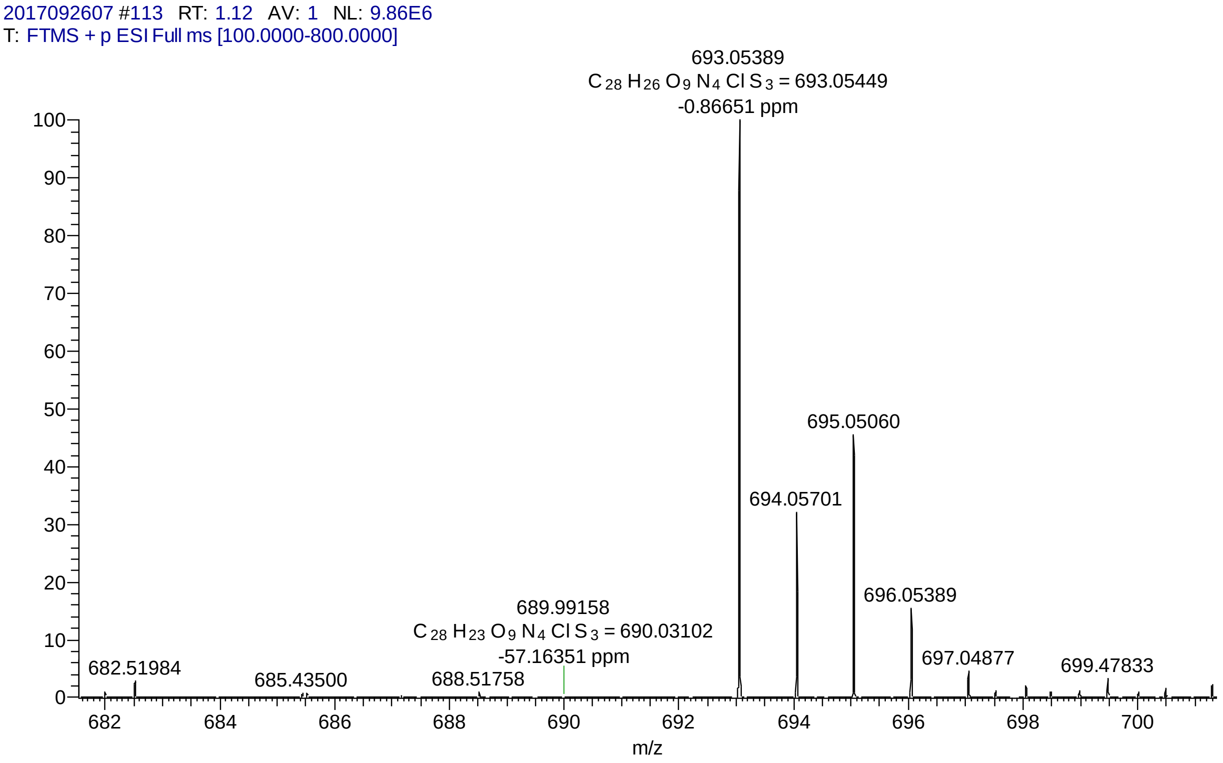

HRMS of compound **5o**

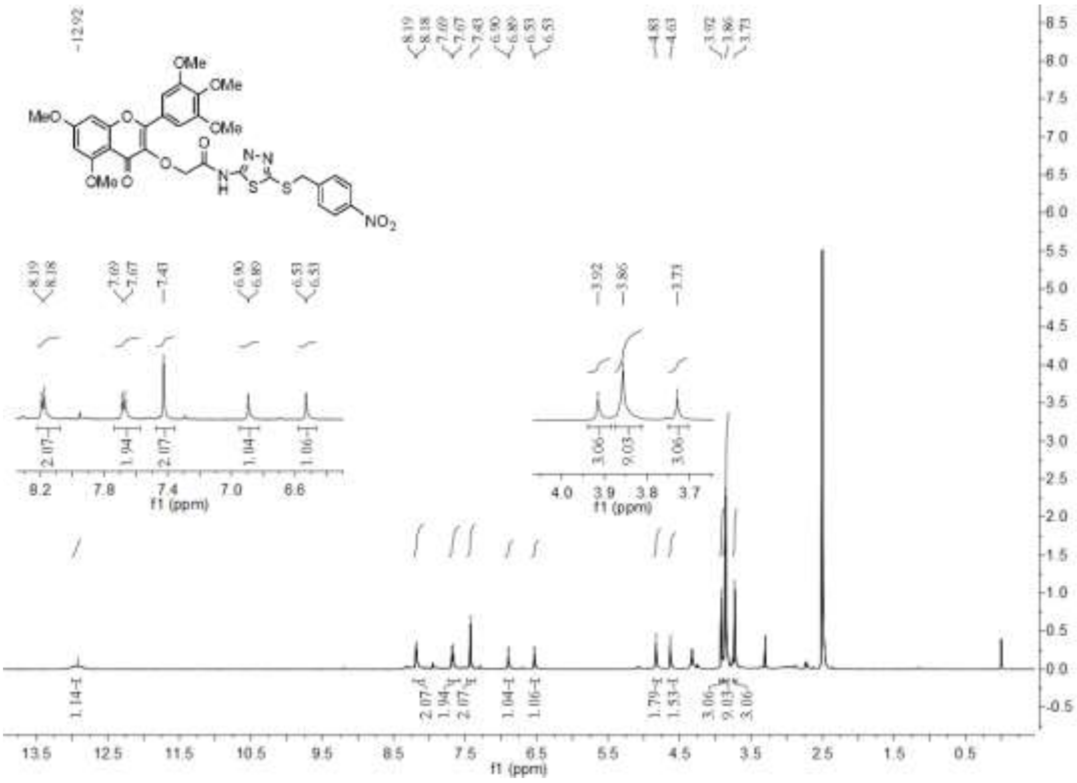

^{1}H NMR of compound **5p**

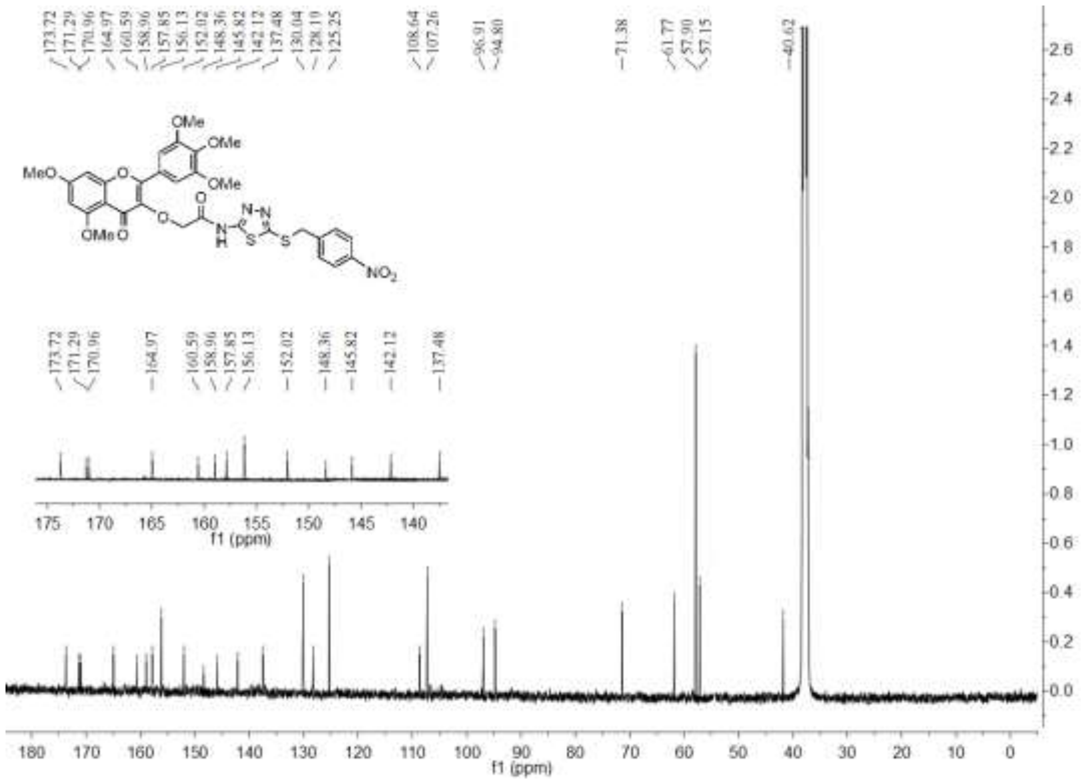

^{13}C NMR of compound **5p**

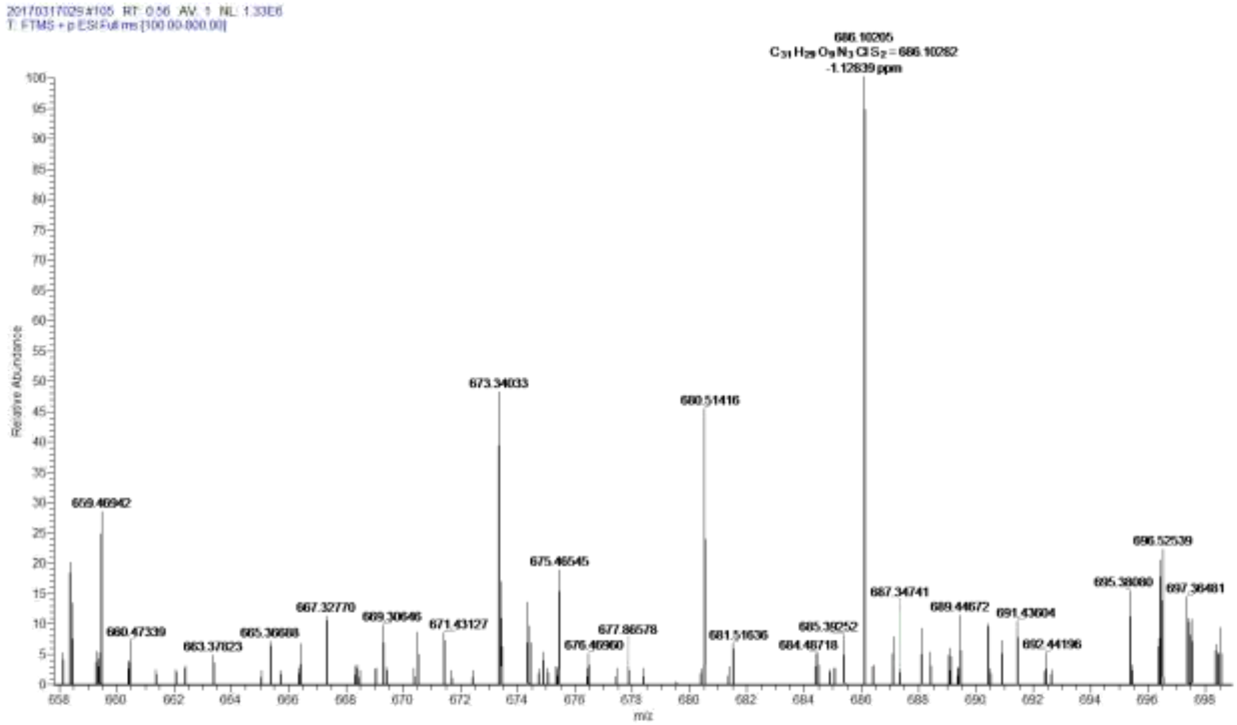

HRMS of compound **5p**
